# The Role of m6A Regulator-Mediated Methylation Modification and Tumor Microenvironment Infiltration in Glioblastoma Multiforme

**DOI:** 10.3389/fcell.2022.842835

**Published:** 2022-02-21

**Authors:** Liang Wang, Haiyan Cao, Ying Zhong, Peigang Ji, Fan Chen

**Affiliations:** ^1^ Department of Neurosurgery, Tangdu Hospital of Fourth Military Medical University, Xi’an, China; ^2^ Department of Biochemistry, University of Greifswald, Greifswald, Germany

**Keywords:** m6A, methylation, TME, GBM, novel immunotherapy, pan-cancer

## Abstract

N6-methyladenosine (m6A) RNA methylation is an emerging epigenetic modification in recent years and epigenetic regulation of the immune response has been demonstrated, but the potential role of m6A modification in GBM tumor microenvironment (TME) cell infiltration and stemness remain unknown. The m6A modification patterns of 310 GBM samples were comprehensively evaluated based on 21 m6A regulators, and we systematically correlated these modification patterns with TME cell infiltration characteristics and stemness characteristics. Construction of m6Ascore to quantify the m6A modification patterns of individual GBM samples using a principal component analysis algorithm. We identified two distinct patterns of m6A modification. The infiltration characteristics of TME cells in these two patterns were highly consistent with the immunophenotype of the GBM, including the immune activation differentiation pattern and the immune desert dedifferentiation pattern. We also identified two modes of regulation of immunity and stemness by m6A methylation. Stromal activation and lack of effective immune infiltration were observed in the high m6Ascore subtype. Pan-cancer analysis results illustrate a significant correlation between m6AScore and tumor clinical outcome, immune infiltration, and stemness. Our work reveals that m6A modifications play an important role in the development of TME and stemness diversity and complexity. Patients with a low m6AScore showed significant therapeutic advantages and clinical benefits. Assessing the m6A modification pattern of individual tumors will help enhance our knowledge of TME infiltration and stemness characteristics, contribute to the development of immunotherapeutic strategies.

## 1 Introduction

In recent years, researchers have identified many novel RNA modifications, including 5-methylcytosine, N1-methyladenosine and N6-methyladenosine (m6A) (Roundtree et al., 2017) The most prominent example is m6A, which is considered to be the most frequent, common and conserved internal modification in eukaryotic cells (Zhang et al., 2020). m6A is involved in regulating gene expression by affecting transcriptional stability (Geula et al., 2015), processing (Ma et al., 2017), splicing (Dominissini et al., 2012), cap-independent translation and translation efficiency (Boccaletto et al., 2018), in addition to promoting cRNA translation (Roignant and Soller, 2017). The m6A modification is a dynamic and reversible process whose function is regulated by binding proteins, demethylases and methyltransferases, namely the readers, erasers and writers of m6A (Yang et al., 2018). They play different functions, the reader mainly affects the recognition process, the eraser affects the consumption, and the writer affects the accumulation. Some m6A regulators (Ms) have been shown to affect multiple biological functions *in vivo* (Qin et al., 2020). In addition, abnormal expression of some Ms was also suggested to be associated with tumorigenesis, progression, abnormal immune regulation and impaired self-renewal ability (Tang et al., 2020).

Glioblastoma multiforme (GBM) is the most common and most aggressive malignant tumor of the central nervous system in adults (Pan et al., 2021b). Nowadays, in the treatment of GBM, the technique of resection, radiation therapy, and chemotherapeutic drugs have made progress, but survival has not been significantly prolonged (Yu et al., 2020). People who have GBM exhibit complex immune dysfunction states, involving multiple mechanisms of local, regional, and systemic immunosuppression and tolerance, immunotherapy has become a new therapeutic approach for GBM. As a current highly promising RNA modification, m6A methylation has been experimentally confirmed to play a key role in the development of various cancers (Vu et al., 2017), while the study of m6A methylation provides a new perspective for GBM treatment. m6A modifications have been shown to potentially affect the formation of a variety of peripheral tumor microenvironments (TME), be involved in cancer stem cells (CSCs) generation and maintenance, the control of cancer progression, and treatment resistance (Ma and Ji, 2020). Previous studies have confirmed that GBM is more heterogeneous than other peripheral tumors, suggesting that multiple factors may influence developmental plasticity and immune checkpoint blockade therapy in GBM, possibly including the TME, RNA modifications, and stem cell phenotype (Malta et al., 2019).

Immunotherapies represented by anti-PD-1 and PD-L1 are currently showing impressive clinical efficacy in a small group of patients. It has been shown that the eraser FTO acts as an m6A demethylase to promote melanoma tumorigenesis and induce resistance of tumor cells to anti-PD-1 therapy, and moreover, the combination of FTO inhibition and anti-PD-1 blockade may synergistically reduce melanoma resistance to immunotherapy (Yang et al., 2019). Another study confirmed that METTL3 and METTL14 significantly promote human GSC growth and tumorigenesis, and that overexpression of METTL3 or inhibition of FTO inhibits GSC self-renewal and growth (Cui et al., 2017). There is also an interaction between Ms, with knockdown of FTO increasing m6A methylation in key pro-tumor cell-intrinsic genes including PD-1, SOX10 and CXCR4, resulting in increased RNA decay through the reader YTHDF2 (Yang et al., 2019). The relationship between some Ms and oncogenic pathways has also been explained (Li et al., 2017). In addition, as an important part of cancer research, several recent studies have revealed a specific association between m6A modifications and TME-infiltrating immune cells (ICs). For example, YTHDF1 increased the translation efficiency of lysosomal cathepsins in dendritic cells (DCs), and inhibition of YTHDF1 improved the therapeutic effect of anti-PD-L1. m6A of the METTL3-mediated TLR4, CD80, and CD40 signaling adaptor Tirap transcripts increased their translation in DCs, thereby stimulating T cell activation and enhancing TLR4/NF-κB signaling-induced cytokine production (Wang et al., 2019). Cancer formation is the result of multiple genes acting together, and previous studies have been limited to one or two Ms and cell types. Thus, it is very necessary to conduct a comprehensive analysis of the expression of all Ms in GBM, including TME ICs infiltration, stemness, and novel immunotherapy, which will provide new theoretical support for subsequent biological studies.

In our study, we collected genes from two databases, TCGA-GBM and CGGA-GBM, to analyze m6A modification patterns and finally determined the molecular, immunological and stemness characteristics of GBM cells with different m6A modification patterns, respectively. Here we identified two different m6A modification patterns for the immune activation/desert dedifferentiation phenotype. With the aim of comparing individual differences in different modifications, we designed the m6AScore system with reference to a previous study. This system was subsequently shown to be closely associated with the prognostic and molecular characteristics of GBM. Moreover, our m6AScore system could predict patient response to novel immunotherapies, and we subsequently further validated the correlation of m6AScore with ICs infiltration and novel immunotherapies in a pan-cancer analysis.

## 2 Materials and Methods

### 2.1 GBM Dataset Source and Preprocessing

Supplementary Figure S1A showed the flow chart of our whole study. The GBM data were derived from two datasets, TCGA-GBM (https://portal.gdc.cancer.gov/) and CGGA-GBM (http://www.cgga.org.cn/). We also collected healthy human data from the Genotype-Tissue Expression (GTEx) database (http://commonfund.nih.gov/GTEx/). 154 methylation data from the Xena repository (https://xena.ucsc.edu/). The dataset used for copy number variation (CNV) analysis was also downloaded from the UCSC Xena browser (https://xenabrowser.net). All of our data were analyzed with the R (version 3.6.1) and R Bioconductor packages.

### 2.2 Unsupervised Clustering of 21 Ms

By reviewing the literature (Chen et al., 2019; Pan et al., 2021a) and extracting from the datasets, we selected a total of 21 widely accepted and reported Ms. The 21 selected Ms include 11 readers (YTHDF1, YTHDF2, YTHDF3, YTHDC1, YTHDC2, IGF2BP1, HNRNPA2B1, HNRNPC, FMR1, LRPPRC, ELAVL1), 8 writers (METTL3, METTL14, WTAP, RBM15, RBM15B, ZC3H13, CBLL1, ZC3H13) and two erasers (ALKBH5, FTO).

### 2.3 Bioinformatic Analysis: Gene Set Variation Analysis and Gene Ontology Annotation

We used ConsensusClusterPlus of the R package to divide TCGA-GBM and CGGA-GBM patients into two clusters each. Principal component analysis (PCA) in each cluster was performed to observe the distribution of gene expression. Differential analysis was performed for each gene in the pre-classified samples using the limma package and the DESeq2 package in R, respectively, and the intersection of the results of the two packages was taken as the differentially expressed genes (DEGs).

Variation in biological processes (BP) between different m6A modification patterns was investigated using Gene Set Variation Analysis (GSVA) analysis. Upregulated DEGs analysis was performed by Gene Ontology (GO) functional analysis and Kyoto Encyclopedia of Genes and Genomes (KEGG) pathway enrichment analysis. The functions associated with different GBM clusters were studied by Gene Set Enrichment Analysis (GSEA).

### 2.4 Estimation of Tumor Microenvironment Cell Infiltration

We used the single-sample gene set enrichment analysis (ssGSEA) algorithm to quantify the relative abundance of each cell infiltrate in the GBM TME. By calculating the enrichment score we can know the relative abundance of TME infiltrated cells in the relative samples, where the minimum value was 0 (Supplementary Table S1). And we classified the immune cells according to their cell functions, the pro-tumor immune cells and immunosuppressive cells include: pDC, Neutrophil, CD56dimNK, TAM, imDC, Th2, MDSC, and Treg; the anti-tumor immune cells include: NKT, TemCD4, TemCD8, ActCD4, ActCD8, Th1, Th17, ActDC, TcmCD4, TcmCD8, CD56briNK, and NK. In addition, we used 29 immune signatures to detect immunophenotypes (Supplementary Table S2). The deconvolution method CIBERSORT (http://cibersort.stanford.edu/) was used to estimate the abundance of 22 different leukocyte subsets with GBM gene expression profiles. To investigate the association between m6A gene features and some related biological pathways, we collected gene sets storing genes associated with a number of BP (Supplementary Table S3) (Subramanian et al., 2005), and 10 sets of oncogenic pathway gene sets (Supplementary Table S4) (Sanchez-Vega et al., 2019).

### 2.5 Identification of Hub Genes Between m6A Different Clusters

Weighted co-expression network analysis of DEGs between the two clusters in the TCGA-GBM cohort was performed using weighted correlation network analysis (WGCNA) package in R to further identify stemness and immunophenotype-related genes. Based on the characterization indices, mRNAsi, ESTIMATE score and mDNAsi and were defined as interesting phenotypes for further studies. To further explore the relationship between each module Eigengenes and the clinical parameters, hypergeometric tests were used to design the overlap between the parameters and the combined modules, and the correlation between the parameters and the module Eigengenes expression pattern was used as gene significance (GS).

### 2.6 Construction of a Scoring System That Can Quantify m6A Gene Signature

In order to quantify the m6A modification patterns of individual tumors and assess their impact on sensitivity to different treatments, we constructed a scoring system, called m6Ascore. The procedures for building m6AScore are as follows:

The DEGs identified from two clusters of the TCGA-GBM cohort were normalized and the crossover genes between the two clusters were extracted, the procedure was repeated in the CGGA-GBM cohort, and finally, the overlapping genes of the two crossover results were extracted. Prognostic analysis was performed for each gene in the signature using a univariate Cox regression model, and genes with prognostic significance were extracted for final analysis. And then we performed PCA to construct an m6A-associated gene signature, and principal components 1 and 2 were both selected as feature scores. With the principal component results, we used a method similar to the gene expression grade index (Sotiriou et al., 2006) formula to construct the m6Ascore:

$$m6\mathrm{Ascore}=\left[\sum \left(\mathrm{PC}1_{g}+\mathrm{PC}2_{g}\right)-X\right]/\mathrm{SD}$$

g indicates the expression of m6A phenotype-related genes. X indicates the average of PC1g + PC2g values of all samples in the cohort. SD indicates standard deviation.

### 2.7 Assessment and Validation of the Ability of the m6A Scoring System to Reflect Treatment Sensitivity in Different Malignant Tumors

To analyze the association of m6AScore with immunotherapy, we collected three cohorts of patients with metastatic melanoma treated with anti-PD-1 therapy (Hugo et al., 2016), patients with metastatic uroepithelial cancer treated with anti-PD-L1 therapy (Mariathasan et al., 2018), patients with GBM treated with anti-PD-1 therapy (Lee et al., 2021) (Cloughesy et al., 2019). To investigate the differences in the extent to which m6A modifications affect immunity and stemness, immune markers and typical BP were introduced to compare the potential mechanisms of the different clusters. We used the TCGAmutations package in R to calculate the TMB.

### 2.8 Statistical Analysis

All statistical analyses in our study were generated by R (version 3.6.1). For comparisons between two groups, the chi-square test was used, and for comparisons among three or more groups, one-way ANOVA and the Kruskal–Wallis test were used as parametric and non-parametric methods, respectively. Spearman and distance correlation analyses were performed to calculate correlation coefficients between sample phenotypes and expression of Ms and m6AScores. Survival curves for all prognostic analyses in this study were performed by the Kaplan-Meier method, and the significance of differences was determined using the log-rank test. hazard ratios for m6A phenotype-associated genes and Ms were calculated by the univariate Cox regression model, and the remaining independent prognostic factors were determined by multivariate Cox regression models. The standardization method used to normalize the multi-omics data is the scale function in R. The forestplot package in R was used to visualize the results of univariate and multivariate prognostic analyses of m6AScores and other clinical parameters.

## 3 Results

### 3.1 The Landscape of Genetic Variation of 21 m6A Regulators in GBM

**FIGURE 1:**
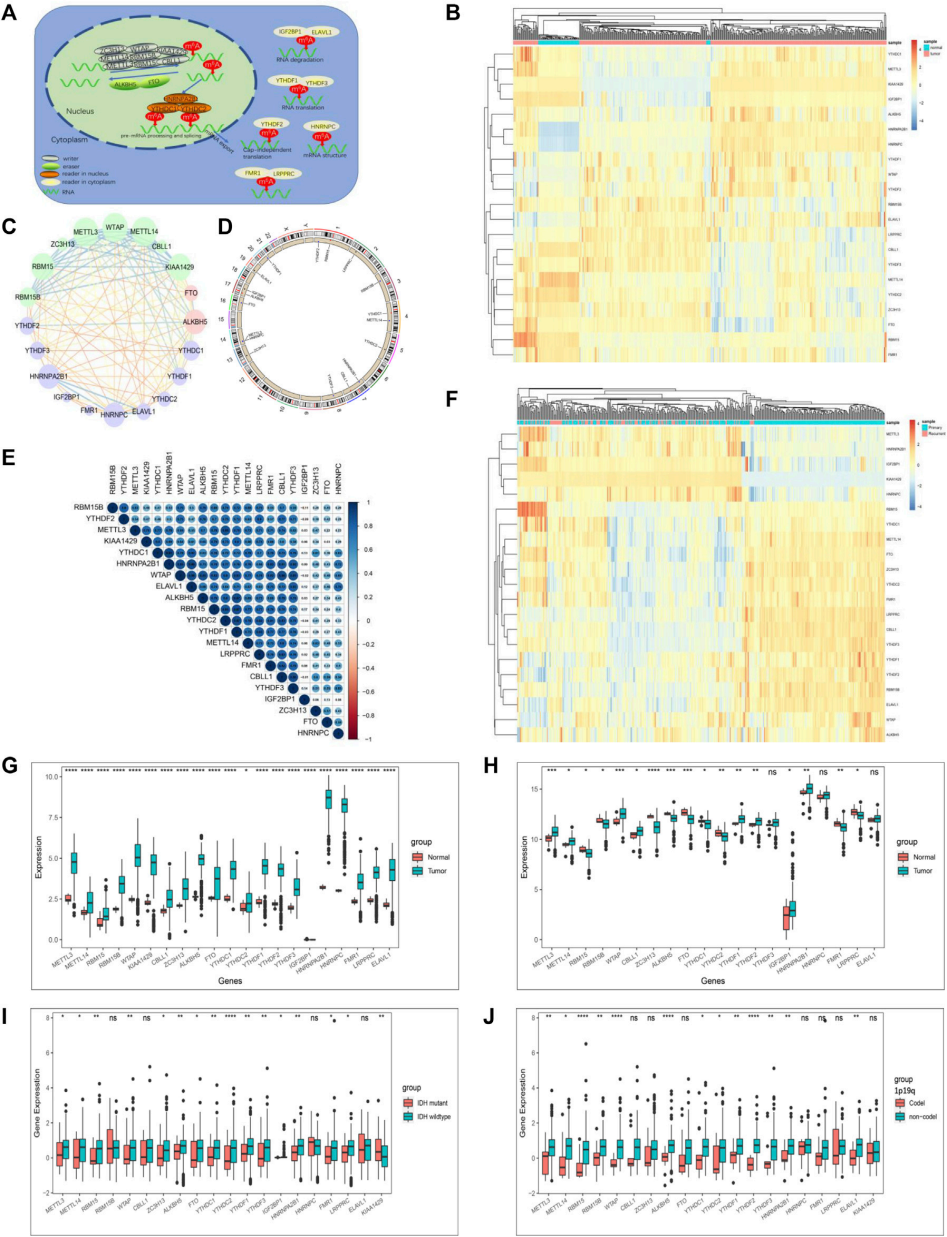
The landscape of genetic variation of m6A regulators in GBM. **(A)** Reversible and dynamic processes of m6A RNA methylation mediated by 21 regulators and their potential biological functions for RNA. **(B)** Heatmaps of expression levels of 21 m6A RNA methylation regulators. Pink represents GBM samples, green represents normal samples. **(C)** A summary of protein-protein interactions between 21 m6A regulators. The lines linking regulators showed their interactions, the circle size represented the connection strength of each node. Erasers were labeled as pink dots in the circle; writers were labeled as green dots in the circle; readers were labeled as blue dots in the circle. **(D)** The location of CNV alteration of 21 m6A regulators on 23 chromosomes. The red dot in the outer ring indicates amplification, while the blue dot in the inner ring indicates deletion. **(E)** Heatmaps of expression levels of 21 m6A RNA methylation regulators from 423 samples. Pink represents recurrent samples, green represents primary samples, columns represent samples, rows represent regulators. **(F)** Spearman correlation analysis of the studied m6A modification regulators. **(G)** The expression of m6A regulators between normal tissues and GBM tissues in the CGGA-GBM cohort. **(H)** The expression of m6A regulators between normal tissues and GBM tissues in the TCGA-GBM cohort. **(I)** The expression of m6A regulators between IDH molecular subtypes. **(J)** The expression of m6A regulators between 1p/19q subtypes. The upper and lower ends of the boxes represented an interquartile range of values. The lines in the boxes represented the median value, and the dots showed outliers. The asterisks represented the statistical *p*-value (*p < 0.05; **p < 0.01; ***p < 0.001, ns, no significant).

We summarize the dynamic reversible process of these m6A RNA methylations mediated by Ms, including the identification, addition and removal of m6A modification sites, and their biological functions on RNA (Figure 1A). We evaluated the association between the Ms and the clinical molecular phenotype of GBM, and Figure 1B revealed that the methylation levels of Ms were different between GBM and normal tissues. We used the Ms network to paint a comprehensive landscape of Ms interactions and regulator connections (Figure 1C). We identified that not only Ms in the same category showed a significant correlation in expression, but also Ms in different categories showed a significant correlation with each other. METTL3 and METTL14 were the hub nodes of the m6A writers, and the hub node of the reader was HNRNPA2B1. Recently, He et al. (He et al., 2021) demonstrated that METTL3 regulates tumor growth by cooperating with YTHDF2. This is to some extent a validation of our prediction. We labeled the locations of Ms showing amplification or deletion of individual regulators and compared the differences in CNV of individual Ms in normal and GBM tissues and found significant differences in 4 Ms, and these results suggested that the CNV status of these 4 Ms correlates with the development of GBM (Figure 1D). Further correlation analysis of regulator co-expression revealed significant correlations between YTHDC1, KIAA1429, HNRNPA2B1 and multiple Ms, with the highest correlation coefficient between YTHDC1 and HNRNPA2B1 (0.91) (Figure 1E). We further evaluated the difference in expression of methylation levels of Ms between primary and recurrent GBM tissues (Figure 1F). In addition, we analyzed the relationship between writers, readers and erasers. There were significant differences in the expression values of 17 and 12 Ms in the high versus low FTO expression group, and 17 and 15 Ms in the high versus low ALKBH5 group, respectively (Supplementary Figures S1D–G, S2A–D).

The correlation between the molecular characteristics of GBM and the expression patterns of Ms was next investigated. The difference in Ms expression in normal versus GBM tissues showed statistical significance (Figures 1G,H). Although Ms’ expression showed differences between primary and recurrent GBM, this difference was not statistically significant (Supplementary Figures S1B,C). In addition, we found that the expression values of most of the Ms were significantly different in the groups based on molecular subtype classification (Figure 1I), 1p19q classification (Figure 1J), TCGA-GBM subtypes (Supplementary Figure S3).

To analyze the prognostic value of Ms in the GBM cohort, we used Cox proportional hazards regression analyses employing univariate and multivariate models. The results showed that five moderators were significantly associated with overall survival (OS) in the univariate model and four moderators were significantly associated with OS in the multivariate model (Supplementary Figures S2E,F). We also investigated the correlation between the expression values of Ms and the composition of ICs and immune signatures (Supplementary Figures S2G,H). ALKBH5, WTAP, RBM15B, FTO, YTHDF1, YTHDF2, YTHDF3, LRPPRC, and ELAVL1 were significantly and positively correlated with immune signatures and ICs composition.

### 3.2 Identification of Two Clusters of GBM Samples

**FIGURE 2:**
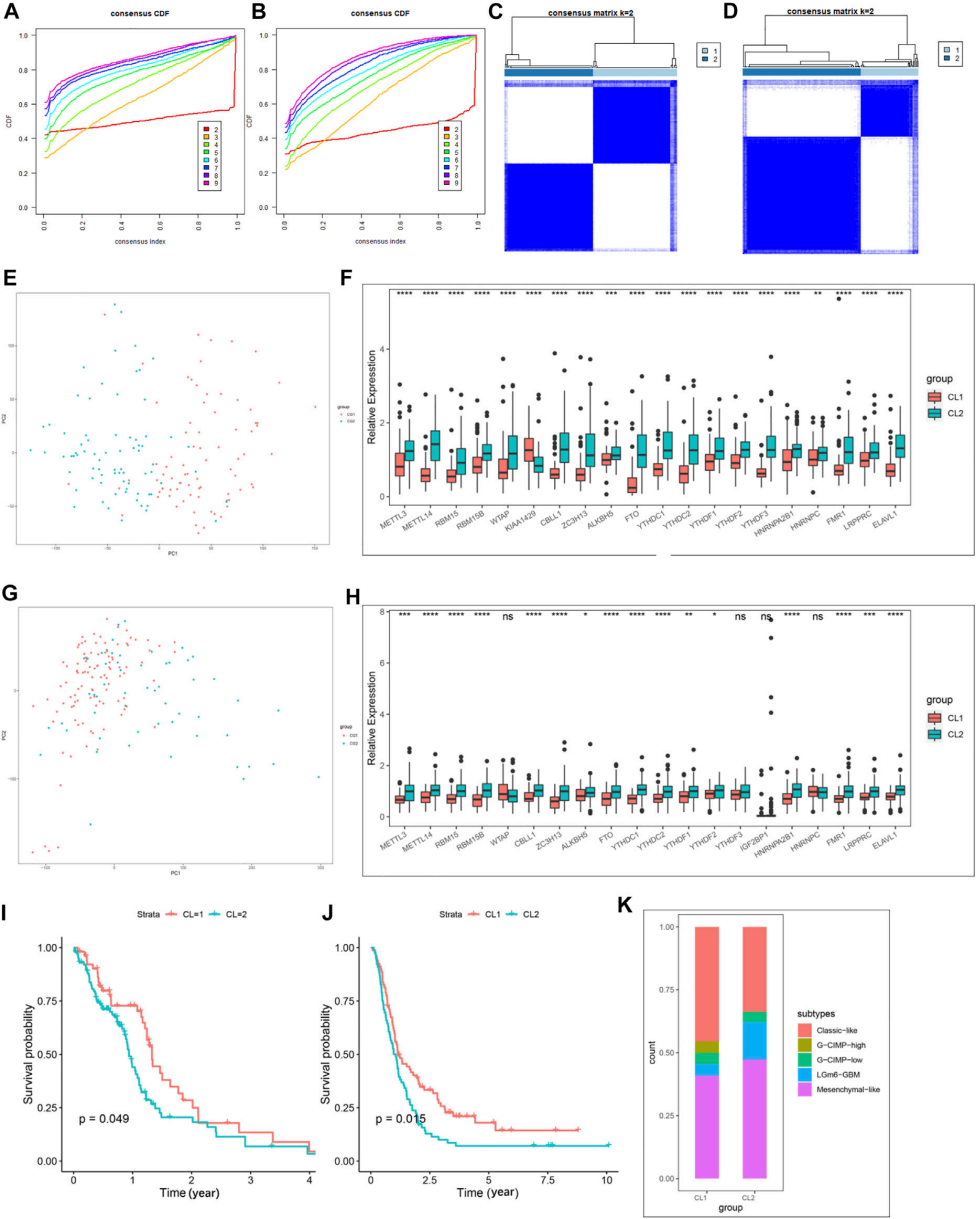
Differential clinicopathological characteristics and overall survival of GBM in the CL1/2 subgroup. **(A)** Consensus clustering CDF for k = 2–10 in CGGA-GBM cohort. **(B)** Consensus clustering CDF for k = 2–10 in TCGA-GBM cohort. **(C)** Consensus clustering matrix for k = 2 in the CGGA-GBM cohort. **(D)** Consensus clustering matrix for k = 2 in the TCGA-GBM cohort. **(E)** Principal component analysis of the total mRNA expression profile in the CGGA-GBM cohort. **(F)** The expression of 21 m6A regulators between the CL1 and CL2 groups in the CGGA-GBM cohort. **(G)** Principal component analysis of the total mRNA expression profile in the TCGA-GBM cohort. GBM patients in the CL1 subgroup are marked with red, GBM patients in CL2 are marked with blue. **(H)** The expression of 21 m6A regulators between the CL1 and CL2 groups in the TCGA-GBM cohort. **(I)** Kaplan–Meier overall survival curves for different CL1 and CL2 groups in TCGA-GBM cohort. **(J)** Kaplan–Meier overall survival curves for different CL1 and CL2 groups in CGGA-GBM cohort. **(K)** The proportions of DNA methylation subtypes identified by supervised clustering in the CL1 or CL2 groups. The upper and lower ends of the boxes represented an interquartile range of values. The lines in the boxes represented the median value, and the dots showed outliers. The asterisks represented the statistical *p*-value (*p < 0.05; **p < 0.01; ***p < 0.001, ns, no significant).

We then extracted GBM samples with complete clinical parameters from the TCGA-GBM and CGGA-GBM cohorts separately for subsequent consensus clustering analysis. From the sample size of both cohorts, when k = 3, unbalanced distributions were observed in all three subgroups of the CGGA-GBM (Supplementary Figure S4A) and TCGA-GBM (Supplementary Figure S4B) cohorts. By combining the relative changes in the area under the consensus clustering cumulative distribution function (CDF) curve for k = 2–10 (Supplementary Figures S4C,D), and the changes in the CDF (Figures 2A,B), we concluded that k = 2 (Figures 2C,D) was the best choice for expressing similarity based on the m6A regulator. Next, the GBM samples were pre-classified into two groups by consensus clustering analysis (Supplementary Tables S5, S6). Our results from PCA of the two clusters demonstrated a significant difference in transcriptional profiles between the CL1 and CL2 groups (Figures 2E,G). Based on the significant differences in PCA analysis, we further analyzed the differences in Ms expression between the two clusters, and the results demonstrated that the expression values of most of the Ms presented significant differences between CL1 and CL2 groups (Figures 2F,H). Survival curves from K-M survival analysis for the CL1 and CL2 subgroups showed significantly lower OS in the CL2 compared to the CL1 subgroup (Figures 2I,J). The proportions of DNA methylation subtypes, molecular subtypes, and TCGA subtypes in the CL1 and CL2 subgroups are shown in Figure 2M; Supplementary Figures S4E,F. We can see that the samples with IDH mutation, classical or mesenchymal subtypes are mainly distributed in the CL2 group. This result was consistent with the report that classical and mesenchymal have a worse prognosis than neural and proto-neural (He et al., 2021). The results of the above study confirmed that consensus clustering results were closely related to the prognosis of GBM.

### 3.3 Annotation of Classification Functions Determined by Consensus Clustering Analysis

**FIGURE 3:**
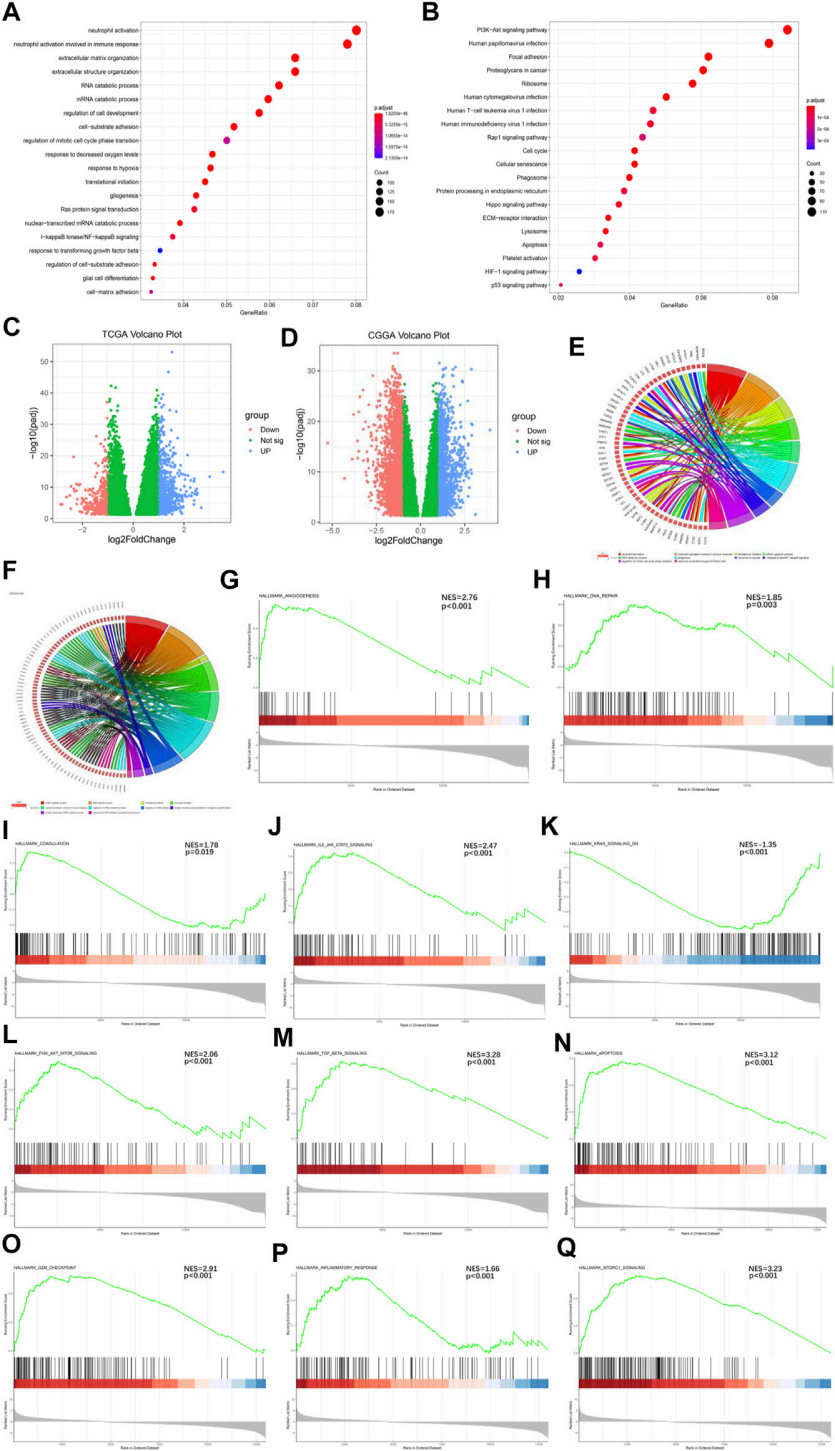
Interaction among functional annotation of GBM and 21 m6A RNA methylation regulators in CL1/2 subgroups. **(A)** Functional annotation for m6A-related genes using GO enrichment analysis in TCGA-GBM cohort. **(B)** Functional annotation for m6A-related genes using KEGG enrichment analysis in TCGA-GBM cohort. **(C)** Interaction among 21 m6A RNA methylation regulators and functional annotation of GBM in CL1/2 subgroups in TCGA cohort. **(D)** Interaction among 21 m6A RNA methylation regulators and functional annotation of GBM in CL1/2 subgroups in CGGA cohort. The red dots represent downregulated DEGs, the green dots represent non-regulated DEGs, and the blue dots represent upregulated DEGs. **(E)** Functional annotation of the upregulated DEGs in the CL2 subgroup of GO analysis in TCGA cohort. **(F)** Functional annotation of the upregulated DEGs in the CL2 subgroup of GO analysis in CGGA cohort. **(G–Q)** GSEA revealed that genes with higher expression in the CL2 subgroup were enriched for hallmarks of malignant tumors, Angiogenesis in TCGA-GBM cohort **(G)**, DNA repair in TCGA-GBM cohort **(H)**, Coagulation in TCGA-GBM cohort **(I)**, IL6-JAK-STATS signaling in TCGA-GBM cohort **(J)**, Kras signaling in TCGA-GBM cohort **(K)**, PI3k-AKT-MTOR signaling in TCGA-GBM cohort **(L)**, TGFβ in TCGA-GBM cohort **(M)**, Apoptosis in CGGA-GBM cohort **(N)**, G2M-Checkpoint in CGGA-GBM cohort **(O)**, Inflammatory response in CGGA-GBM cohort **(P)**, MTORC1 signaling in CGGA-GBM cohort **(Q)**.

To further understand the mechanisms by which the Ms affect GBM progression, we investigated DEGs between CL1 and CL2 subgroups. By differential analysis, a total of 1,000 genes were identified as DEGs in the TCGA-GBM cohort (Figure 3C) and 3,948 genes were identified as DEGs in the CGGA-GBM cohort (Figure 3D). To investigate the potential functions of DEGs, we performed KEGG pathway analysis and GO functional analysis on 634 and 1,576 significantly upregulated genes in the CL2 subgroups of TCGA-GBM (Figures 3A,B; Supplementary Table S7) and CGGA-GBM (Supplementary Figures S4G,H; Supplementary Table S8), respectively. The top 10 GO terms for TCGA-GBM indicated that upregulated genes were enriched in neutrophil activation, neutrophil activation involved in immune response, translational initiation, mRNA catabolic process, gliogenesis, response to hypoxia, I-κB kinase/NF-κ B signaling, regulation of mitotic cell cycle phase transition, response to transforming growth factor β. The top 10 GO terms for TCGA-GBM and CGGA-GBM (Figures 3E,F) indicated that upregulated genes were enriched in malignancy associated processes, including neutrophil activation, neutrophil-mediated immunity, translational initiation, mRNA and RNA catabolic process, cell proliferation, cell-substrate adhesion, response to hypoxia, response to transforming growth factor β and I-κB kinase/NF-κ B signaling.

Meanwhile, our GSEA analysis showed that the malignant hallmarks of tumors, including Angiogenesis, DNA repair, Coagulation, IL-6/JAK/STAT3 signaling, KRAS signaling, PI3K/AKT/MTOR signaling, TGFβ signaling were significantly related to the CL2 subgroup in TCGA-GBM cohort (Figures 3G–M). In the analysis of the CGGA-GBM cohort, CL2 subgroup was statistically significant associated with the following markers in addition to the above malignant hallmarks of tumors (Supplementary Figures S4I–O), including Apoptosis, G2M checkpoint, Infammatory response, MTORC1 signaling (Figures 3N–Q).

### 3.4 TME Immune Cell Infiltration Characteristics in Distinct m6A Modification Patterns

**FIGURE 4:**
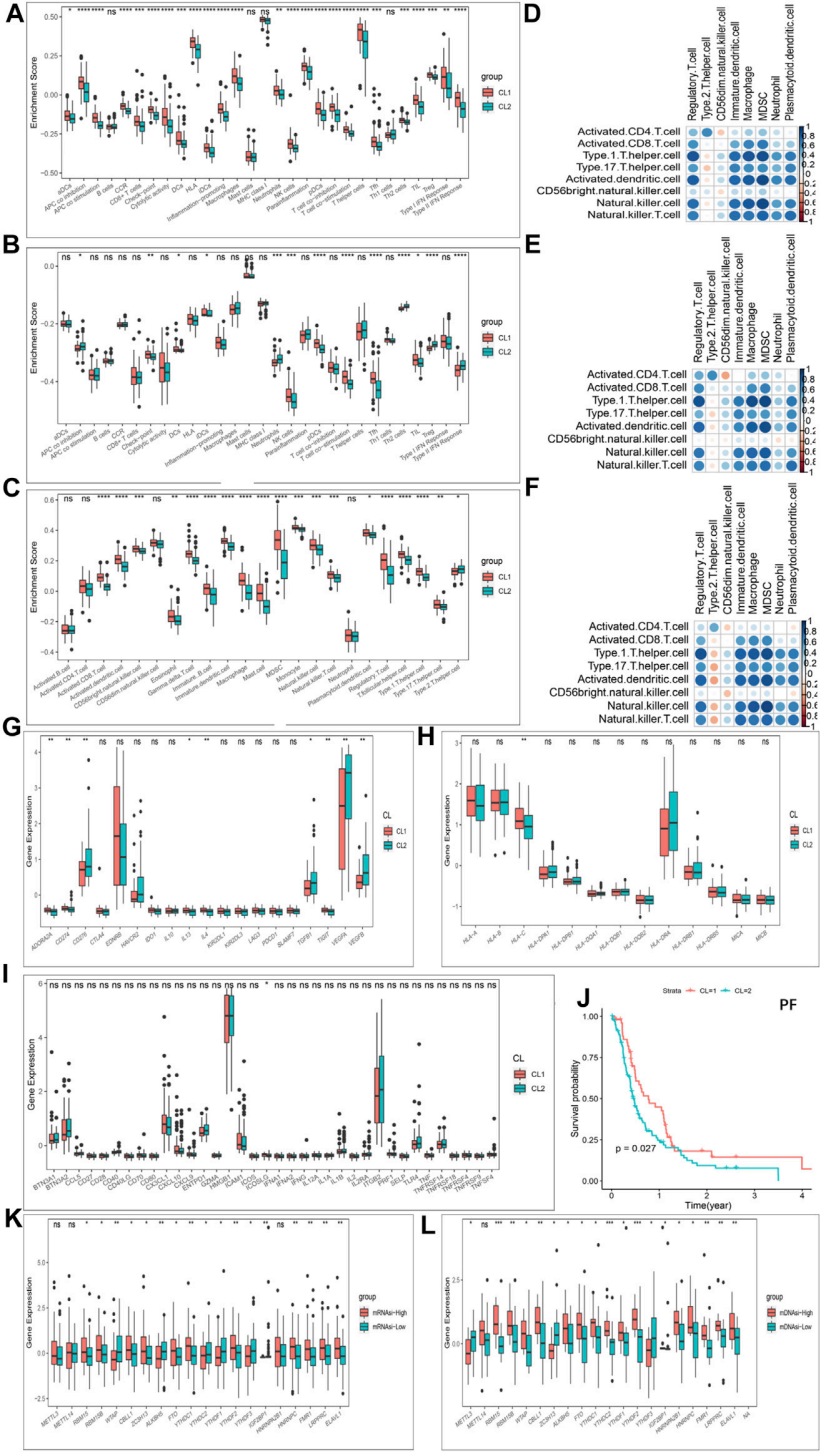
Patterns of m6A methylation modification and biological characteristics of each pattern. **(A)** The enrichment differences of immune signatures between CL1 and CL2 subgroups in the TCGA-GBM cohort. **(B)** The enrichment differences of immune signatures between CL1 and CL2 subgroups in the CGGA-GBM cohort. **(C)** The enrichment fraction differences of immune cells between CL1 and CL2 subgroups in the TCGA-GBM cohort. **(D–F)** Correlation of samples between infiltration of cell types executing pro-tumor, immune-suppressive functions (pDC, Neutrophil, CD56dimNK, TAM, imDC, Th2, MDSC, and Treg) and cell types executing anti-tumor immunity (NKT, TemCD4, TemCD8, ActCD4, ActCD8, Th1, Th17, ActDC, TcmCD4, TcmCD8, CD56briNK, and NK). **(D)** All samples in the TCGA-GBM cohort. **(E)** CL1 group samples in the TCGA-GBM cohort. **(F)** CL2 group samples in the TCGA-GBM cohort. **(G)** Differences in the expression of inhibitor genes in the CL1 and CL2 subgroups of TCGA-GBM cohort. **(H)** Differences in the expression of MHC genes in the CL1 and CL2 subgroups of TCGA-GBM cohort. **(I)** Differences in the expression of stimulator genes in the CL1 and CL2 subgroups of TCGA-GBM cohort. **(J)** Kaplan-Meier curves for progression-free survival for two robust clusters in the TCGA-GBM cohort in the log-rank test. **(K)** Differences in the expression of m6A regulators in the mRNAsi-high and mRNAsi-low subgroups in TCGA-GBM cohort. **(L)** Differences in the expression of m6A regulators in the mDNAsi-high and mDNAsi-low subgroups in TCGA-GBM cohort. The upper and lower ends of the boxes represented an interquartile range of values. The lines in the boxes represented the median value, and the dots showed outliers. The asterisks represented the statistical *p*-value (*p < 0.05; **p < 0.01; ***p < 0.001, ns, no significant).

Furthermore, in immune signatures’ analyses, the CL2 subgroup was downregulated compared to the CL1 subgroup in both pro- and anti-tumor immune signatures (Figures 4A,B; Supplementary Table S9), suggesting that the effect of this modification pattern on tumor immunity is bidirectional. In the study of ICs enrichment in both clusters, we found that the CL1 subgroup exhibited an enrichment advantage for almost all ICs relative to the CL2 subgroup (Figure 4C; Supplementary Figure S4P; Supplementary Table S9), which may be related to the fact that patients in the CL1 subgroup presented a significant survival advantage. We then divided ICs into two main categories including pro- and anti-tumor ICs and compared their relevance. We first investigated the correlation between pro- and anti-tumor ICs in the overall sample (Figure 4D), the CL1 subgroup (Figure 4E) and the CL2 subgroup (Figure 4F) of the TCGA-GBM cohort, and found that the CL1 subgroup had higher anti-tumor immunity, while the CL2 subgroup showed more tumor immune-promoting effects. The results from CGGA-GBM cohort (Supplementary Figures S5A–C) also confirmed it. To further understand the effect of different modification patterns on TME, we analyzed the expression differences of typical immune-related genes in the two clusters. In the TCGA-GBM cohort, a significant increase in the expression of checkpoint inhibitor-related genes was found in the CL2 group compared to the CL1 group (Figure 4G), while the expression of major histocompatibility complex (MHC) (Figure 4H) and stimulator-related genes (Figure 4I) did not show significant differences. To our surprise, the expression of inhibitor (Supplementary Figure S5E) and MHC-related genes (Supplementary Figure S5F) in the CGGA-GBM cohort was found to be similar to the results of the TCGA-GBM cohort, but the expression of stimulant-related genes (Supplementary Figure S5D) showed significant differences in the two clusters. Accordingly, it is reasonable to believe that different DNA damage-related phenotypes in the two clusters may promote or inhibit the anti-tumor capacity of ICs. In view of this, we simultaneously examined which BP were enriched for DNA damage and showed that DNA-related processes, such as Angiogenesis, cell cycle, DNA replication, mismatch repair and WNT target were significantly increased in the CL2 subgroups compared to the CL1 subgroups (Supplementary Figures S4Q,R; Supplementary Table S9).

Another hot topic of research in TME-related studies at GBM is GBM cancer stem cells (GSCs). Recent studies have confirmed the effect of m6A modifications on the state transition and drug resistance of GSCs (Zepecki et al., 2021). As described in the methods section, we established four stemness indices, including mRNAsi, mDNAsi, EREG-mRNAsi and EREG-mDNAsi with reference to the study of Malta et al. (Malta et al., 2019). We then classified the GBM cohort based on four stemness indices and investigated the differences in Ms expression in each stemness index classification group. Figures 4K,L showed that almost Ms were significantly differentially expressed in the mRNAsi-high and -low groups as well as the mDNAsi-high and -low groups. Similar results were also observed between the high and low groups of EREG-mRNAsi and EREG-mDNAsi (Supplementary Figures S5G,H). Progression-free survival (PF) was further analyzed using K-M survival analysis for the CL1 and CL2 subgroups, with the CL1 subgroup being significantly higher than the CL2 group (Figure 4J). We further investigated the difference in somatic mutations distribution between the two clusters, and the tumor TP53 mutational load was higher in the CL2 subgroup than in the CL1 subgroup, 38 and 20%, respectively (Supplementary Figures S5I,J). TP53 expression has been shown to be associated with poorer prognosis in GBM (Kwok et al., 2017).

In terms of immune characteristics, the CL1 subgroup was the immune activating differentiated phenotype exhibiting immune activation and anti-tumor immune infiltration, while CL2 was the immune desert dedifferentiated phenotype exhibiting immune suppression and promoting tumor immune infiltration. In addition, the CL2 subgroup showed dedifferentiation and DNA damage relative to the CL1 subgroup.

### 3.5 Identification of Hub Genes and Functional Annotation

**FIGURE 5:**
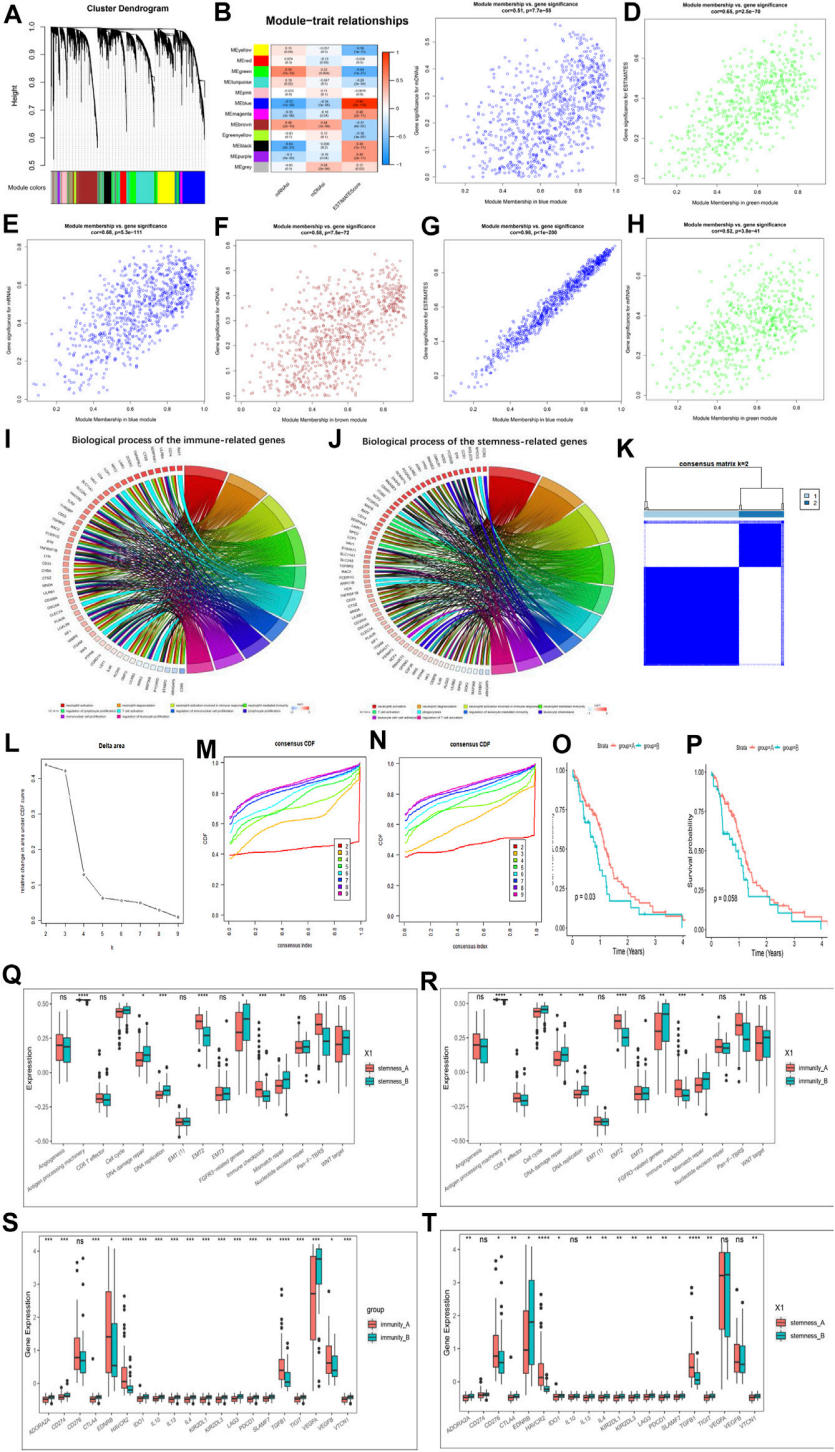
Hub genes’ functional annotation and characteristics of potential traits in m6A-related phenotypes. **(A)** Hierarchical clustering dendrograms of identified co-expressed genes in modules. The branches of the cluster dendrogram correspond to the different gene modules. Each leaf on the cluster dendrogram corresponds to a gene Each colored row represents a color-coded module. A total of 12 merged modules were identified. **(B)** Correlations between the gene modules and clinical traits. The correlation coefficient in each cell represented the correlation between the gene module and the clinical traits, which decreased in size from red to blue. **(C–H)** Scatter plot of module eigengenes in the blue module with mDNAsi **(C)**, a green module with ESTIMATE score **(D)**, a blue module with mRNAsi **(E)**, a brown module with mDNAsi **(F)**, a blue module with ESTIMATE score **(G)**, a green module with mRNAsi **(H)**. Cor is the coefficient indices and *p* is Pearson’s correlation. **(I)** GO Functional annotation of the immunity phenotype-related genes of the biological processes. **(J)** GO Functional annotation of the stemness phenotype-related genes of the biological processes. **(K–M)** Consensus clustering analysis based on the 159 immune phenotype-related genes, **(K)** consensus clustering matrix for k = 2, **(L)** the relative change in area under the CDF curve for k = 2–10, **(M)** consensus clustering CDF for k = 2–10. **(N)** Consensus clustering analysis based on the 159 stemness phenotype-related genes, consensus clustering CDF for k = 2–10. **(O)** Kaplan–Meier curves for two stemness clusters in the Log-rank test. **(P)** Kaplan–Meier curves for two immunity clusters in the Log-rank test. **(Q)** The enrichment of typical biological processes between the two stemness clusters. **(R)** The enrichment of typical biological processes between the two immunity clusters. **(S)** The expression of inhibitor genes among Immunity clusters. **(T)** The expression of inhibitor genes among stemness clusters. The asterisks represented the statistical *p*-value (*p < 0.05; **p < 0.01; ***p < 0.001, ns, no significant).

To further pinpoint specific phenotype-associated genes for every m6A modification pattern, WGCNA was performed to identify biologically significant modules corresponding to the specified phenotype-associated genes. The DEGs used to build a scale-free system were determined by using *p* < 0.05 and |logFC| > 1 as cutoff criteria. Supplementary Figures S6A,B showed our scale-free graph, choosing the most appropriate β value12 to convert the adjacency matrix into a scale-free topology. By combining modules with correlation coefficients above 0.75, we obtained a total of 12 modules (Figure 5A). After that we first identified the module Eigengenes (MEg) represented by the gene expression patterns in the module and then calculated the correlation with the specified phenotypes. Figure 5B showed the key modules associated with specific phenotypes in GBM. The most positively correlated modules for the mRNAsi, mDNAsi and ESTIMATE were MEgreen, MEbrown and Meblue, respectively, and the most negatively correlated modules were MEblue, MEblue, and Megreen, respectively. We first performed further analysis of the hub genes of all modules of mRNAsi (Supplementary Figures S6C–E), ESTIMATE (Supplementary Figures S6F–H), and mDNAsi (Supplementary Figures S7A–C) respectively, further validating our identification of the most positive and most negative modules. Next, we combined the heat map results to select the hub genes in the key modules for additional analysis, and the relevant results were shown in Figures 5C–H.

We then identified a total of 186 immune phenotype-associated genes and 107 stemness phenotype-associated genes (Supplementary Table S10). To our surprise, several genes related to the stemness and immunity phenotype-related genes were overlapped, suggesting that the regulation of tumor immunity and stemness by m6A modifications is not completely independent. Simultaneous GO analysis for genes associated with the immune phenotype and the stemness phenotype showed that both enrichment BP were closely associated with tumor immunity and stemness (Figures 5I,J; Supplementary Table S11).

We next correlated 186 immune phenotype-associated genes and 107 stemness phenotype-associated genes with reference to unsupervised consistent clustering analysis of m6A modification patterns, and divided the total sample into corresponding subgroups. By combining the distribution of samples (Figure 5K; Supplementary Figures S7D–H), relative changes in the area under the consensus clustering CDF curve for k = 2–10 (Figure 5L; Supplementary Figure S7I), and the changes in the CDF (Figures 5M,N), we concluded that k = 2 was the best choice (Supplementary Tables S12, S13). K-M analysis showed that patients clustered in the stemness and immune B group presented a better prognostic advantage than A group (Figures 5O,P). In addition to this we also investigated the difference in expression of Ms between immune and stemness subgroup A and B. The results showed that most of the regulators were significantly differentially expressed between the groups (Supplementary Figures S7J,K).

We next further explored potential trait characteristics associated with both regulatory modes of immunity and stemness, first comparing differences in BP and immune signatures typical of each subgroup. The results showed that the Stemness B subgroup exhibited strong enrichment of cell cycle, DNA damage repair, FGFR3-related genes, DNA replication, mismatch repair and WNT target signaling, while the A subgroup exhibited strong EMT, immune checkpoint and pan-fibroblast TGFβ response signaling pathways (Figure 5Q). Further exploration of the expression of stemness markers between the groups revealed significant differences (Supplementary Figure S7L). In immune groups showed similar patterns of BP enrichment, with immune B subgroup characterized by enrichment of gene-related pathways and A subgroup by enrichment of immune-related pathways (Figure 5R). ICs infiltration was further investigated between the subgroups, and it was found that A subgroup showed a massive infiltration of ICs, including both pro- and anti-tumor cells (Supplementary Figure S8A), and the same results were found in the stemness A subgroup (Supplementary Figure S8B). We further analyzed the expression of typical immune-related genes in different immunity modification pattern groups and found that the expression of most checkpoint inhibitor (Figures 5S,T), MHC-related genes (Supplementary Figures S8C,D) and stimulation (Supplementary Figures S8E,F) were significantly increased in the immune and stemness B group. Unlike previously, MHC features representing the intensity of antigen presentation showed a significant predominance in the immune and stemnes B subgroups, suggesting that antigen presentation is involved in the immune and stemness regulatory processes in the immune and stemness B subgroups. We also summarized the differences in regulator expression between all subgroups using heat maps (Supplementary Figure S9A).

### 3.6 The Role of m6A Modification Pattern in GBM Prognosis

**FIGURE 6:**
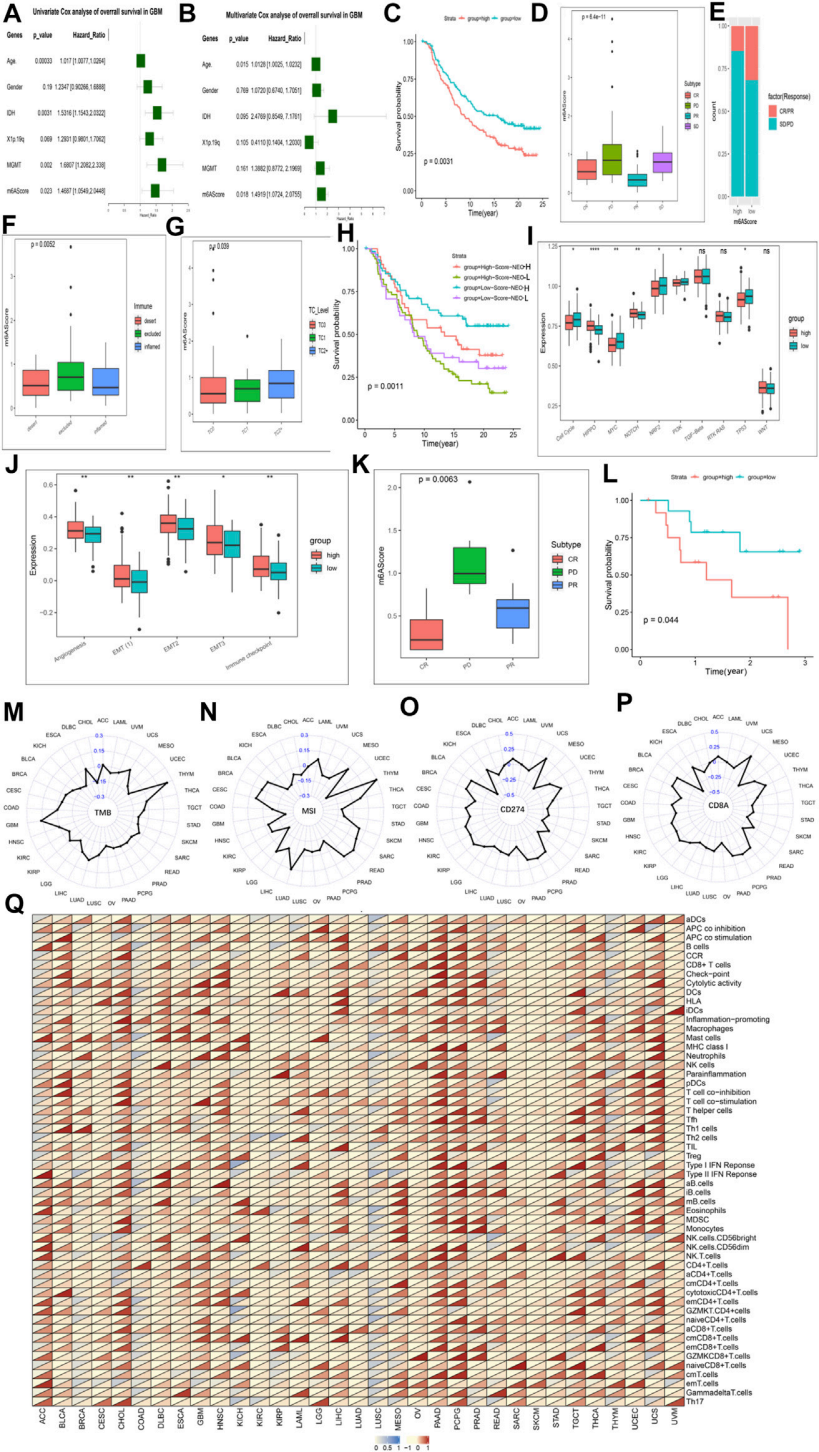
The role of the m6AScore in anti-PD-1/L1 immunotherapy and pan-cancer. Cox regression analyses **(A,B)**. univariate **(A)** and multivariate Cox regression analyses **(B)** of the association between clinicopathological factors (including the m6AScore) and progression-free interval of patients in the TCGA-GBM combined CGGA-GBM cohort. **(C)** Survival analyses for the low-m6AScore and high-m6AScore subgroups in the IMvigor210CoreBiologies cohort (treated with the anti-PD-L1 agent, Atezolizumab) using Kaplan–Meier in Log-rank test. **(D)** The Kruskal–Wallis test detected differences in m6AScore between patients with different clinical responses in the IMvigor210CoreBiologies cohort. **(E)** The proportion of patients in the IMvigor210CoreBiologies cohort with clinical response in the low-m6AScore or high-m6AScore subgroups. **(F,G)** Kruskal–Wallis test detected differences in m6AScore between immunophenotypes **(F)**, tumor cells (TC, **(G)**) in the IMvigor210CoreBiologies cohort. **(H)** Patients receiving anti-PD-L1 immunotherapy were analyzed for survival, stratified by m6AScore and neoantigen load using Kaplan-Meier curves. NEO, neoantigen burden (Log-rank test). **(I)** Differences in 10 oncogenic pathways between low-m6AScore and high-m6AScore subgroups in the IMvigor210CoreBiologies cohort. **(J)** Differences in five biological pathways between low-m6AScore and high-m6AScore subgroups in the IMvigor210CoreBiologies cohort. **(K)** The proportion of patients in the GSE78220 (PD-1) cohort with clinical response in low-m6AScore or high-m6AScore subgroups. **(L)** Survival analyses for the low-m6AScore and high-m6AScore subgroups in the GSE78220 (PD-1) cohort using Kaplan–Meier in Log-rank test. **(M–P)** Radar chart of the correlation between m6AScore and tumor mutation burden (TMB) **(M)**, MSI **(N)** and PD-L1 expression value (CD274) **(O)**, (CD8A) **(P)**. **(Q)** Correlations between the m6AScore and immune cell fractions for each cancer type (Pearson test). A total of 54 gene sets representing distinct immune cell populations was selected, and the ssGSEA scores of each were calculated across 11014 samples in the pan-cancer cohort. The upper and lower ends of the boxes represented an interquartile range of values. The lines in the boxes represented the median value, and the dots showed outliers. The asterisks represented the statistical *p*-value (*p < 0.05; **p < 0.01; ***p < 0.001, ns, no significant).

We obtained the DEGs and constructed m6AScore (Supplementary Table S14). The samples were divided into m6AScore high group and m6AScore low group by using the median of m6AScore as the cut-off value, respectively. We assessed cancer survival between high and low m6AScore groups in different molecular subtypes, different methylation status, 1p19q codeletion, different m6A modification patterns, and TCGA-GBM subtypes using K-M analysis. K-M analysis showed the best survival advantage in the IDH-Mutant-m6AScore low group and the worst overall survival in the IDH-WT-m6AScore high group (Supplementary Figures S9B, S10A), the MGMT-methylated-m6AScore low group had the highest OS and the MGMT-unmethylated-m6AScore high group had the worst OS (Supplementary Figures S9C, S10B). The significant survival advantage of the m6AScore low group over the high group was also observed in the subsequent study of 1p19q (Supplementary Figures S9D, S10C) and m6A modification patterns (Supplementary Figures S9E, S10D). In addition, mesenchymal, neural and proto-neural subtype samples with low m6AScore had survival benefits, while classical subtype hadn’t (Supplementary Figures S10E–H). We performed another separate K-M analysis with the only m6AScore and the results still showed that the OS of the m6AScore low group was significantly higher than that of the high group (Supplementary Figure S9F). Meanwhile m6AScore had a *p* value below 0.05 (HR > 1) in both univariate and multivariate models (Figures 6A,B).

We next explored the mechanisms underlying the prognostic impact of the m6AScore on GBM. First, we analyzed the difference in expression of Ms between the m6AScore high and low groups overall samples, and the results showed that almost Ms were significantly different in expression between the groups (Supplementary Figure S9G). Next, we tested the uniqueness of various m6AScore clusters. We found a higher m6AScore in mesenchymal GBM than in other types, matching the fact that mesenchymal GBM is more malignant than other types of GBM (Hernández-Vega et al., 2020) (Supplementary Figure S10I). Differences in m6AScore were also observed in the immune and stemness clusters, with higher m6AScore in immune and stemness group B than in immune and stemness group B, respectively, which is consistent with the fact that their group B have a worse prognosis (Supplementary Figures S10J,K). We analyzed the differences in ICs infiltration and typical BP between the high and low m6AScore groups and found that most of the infiltrating ICs were significantly enriched in the m6AScore high group (Supplementary Figures S10N,O). But the two cohorts did not show consistent changes in the enrichment of BP (Supplementary Figures S10L,M). We analyzed the enrichment of 10 classical oncogenic pathways (McCubrey et al., 2016) in the high and low m6AScore groups and found that basically all oncogenic pathways were significantly enriched in the high m6AScore group (Supplementary Figure S9H). Correlation analysis of pro- and anti-tumor ICs in the high and low m6AScore groups revealed that pro- and anti-tumor ICs were significantly and positively correlated in all groups, with a greater slope in the low m6AScore group than in the high group, indicating a relatively larger proportion of pro-tumor ICs in the low group (Supplementary Figures S9I,J). Therefore, the tumor immunogenicity difference between the high and low m6AScore groups was significant. We then analyzed the differences in the distribution of somatic mutations between the low and high m6Ascore using the maftools package, and found that mutations in the high m6Ascore group were significantly higher than those in the low group, for example, TP53 was 41% in the high m6Ascore group and 28% in the low group (Supplementary Figures S9K,L).

### 3.7 m6AScore in the Role of anti-PD-1/L1 Immunotherapy

Responsible for cancer immune escape, the PD-1/PD-L1 axis suppresses the immune response and promotes self-tolerance, making a huge impact on cancer treatment (Salmaninejad et al., 2020). Since our our m6AScore system was associated with numerous biomarkers, we decided to study the response of m6AScore to anti-PD-1/PD-L1 therapies and thus identify biomarkers that are sensitive to immunotherapy. We first investigated Ms expression between conventional adjuvant therapy and anti-PD-L1 therapy (IMvigor210), but regulators were not differentially expressed between the two groups (Supplementary Figure S11A). Therefore, novel immunotherapies do not alter the expression of Ms, but this does not mean that the m6AScore does not reflect the sensitivity of immunotherapy. First, we extracted data from three cohorts involving anti-PD-L1 (IMvigor210, urothelial cancer), anti-PD-1 (GSE78220, melanomas) and anti-PD-1 (GSE154795, GSE121810, GBM) immunotherapies, respectively. The drugs involved in the two cohorts were Atezolizumab (anti-PD-L1) and pembrolizumab (anti-PD-1) (Nusrat, 2020), respectively. The m6AScore of the samples of patients treated with anti-PD-L1 was calculated and then subjected to K-M survival analysis, which showed a significant survival advantage for the low m6AScore group over the high group (Figure 6C; Supplementary Table S15). We further analyzed the difference of m6AScore in the sample of patients with urothelial cancer responding to anti-PD-L1 therapy and found that the m6AScore in the response group (CR and PR) was lower than that in the disease group (SD and PD), with a statistically significant difference, indicating that our m6AScore could reflect the sensitivity of patients to anti-PD-L1 therapy (Figure 6D). Moreover, the low m6AScore group was mainly composed of patients from the response group (Figure 6E). We next classified the samples according to the three immunophenotypes and calculated the respective m6AScore, and we found that the immune inflamed phenotype had the lowest m6AScore among the three groups (Figure 6F). This result is consistent with previous reports that inflamed cancers are the most responsive to checkpoint blockade among the three immune phenotypes (Hegde et al., 2016). Moreover, we found that the correlation study of m6AScore and PD-L1 expression on tumor cells (TC) revealed a significant correlation, with TC1 having the lowest m6AScore (Figure 6G). We further investigated the relationship between m6AScore and the number of mutations and showed a negative correlation (Supplementary Figure S11B). Neoantigens are important targets of T cell-mediated antitumor immunity, and tumor neoantigen burden (TNB) can be a direct molecular marker of immunotherapeutic response (Ott et al., 2020), which we used to analyze the survival benefit among patients with m6AScores. K-M analysis showed a negative correlation between m6AScore and TNB, patients with low m6AScore and high TNB having the best survival advantage, while patients with high m6AScore and low TNB had the lowest survival rate (Figure 6H). Finally, we analyzed the enrichment of 10 typical oncogenic pathways and five BP and found that most pathways were highly enriched in the m6AScore-high group (Figures 6I,J; Supplementary Table S16).

In the study of the anti-PD-L1 treatment cohort, it was demonstrated that our m6AScore not only reflects the sensitivity of patients to anti-PD-L1 treatment, but also correlates with the progression of cancer. We next revalidated it in the anti-PD-1 treatment cohort. Similar results were obtained for the response group (CR and PR) and the disease group (PD) (Figure 6K; Supplementary Table S17). Response group samples were also mainly distributed in the low m6AScore group. K-M survival analysis also showed a significant survival advantage for the low m6AScore group (Figure 6L). However, in the analysis of the 10 classical oncogenic pathways, there was no uniform trend between the low and high m6AScore groups, although they showed significant differences (Supplementary Figure S11C), and we considered that it might be an effect of the small sample size. The study of five classical BP got similar results to the anti-PD-L1 treatment cohort, all enriched in the high m6AScore group (Supplementary Figure S11D; Supplementary Table S18).

In the study of the patients with GBM by anti-PD-1 therapy, it also got similar results. Our m6AScore correlates with the progression of GBM. In the analysis of the 10 classical oncogenic pathways, nine of them were enriched in the high m6AScore group (Supplementary Figure S11E). In the study of five classical BP, all of them were enriched in the high m6AScore group (Supplementary Figure S11F). Interestingly, we found that almost all anti-tumor ICswere enriched in the low m6AScore group, nearly all pro-tumor ICs were enriched in the high m6AScore group (Supplementary Figure S11G). We also produced heat maps on each trait based on the m6AScore, showing that different traits were clustered differently in the high and low m6AScore groups (Supplementary Figure S11H).

### 3.8 Expression of Ms in Pan-Cancer and the Utility of m6AScore Across Tumor Types

We first analyzed the expression levels of Ms in the pan-cancer cohort and the results were presented as a heat map (Supplementary Figure S12A). HNRNPA2B1, HNRNPC, LRPPRC, ALKBH5 showed high expression in almost all cancers. Further analysis of the expression differences of 21 Ms between normal and tumor tissues in the pan-cancer database showed that almost all of them were significantly differentially expressed between normal and tumor tissues (Supplementary Figure S12B). CD274 and CD8A (Supplementary Figures S12C,D) showed a broader positive correlation with Ms than GZMA and PRF1 (Supplementary Figures S13A,B) with Ms. CD274 and CD8A can reflect PD-L1 and PD-1 expression values, suggesting that Ms are associated with novel immunotherapy for some cancers.

We have demonstrated a link between m6AScore and immunotherapeutic response, and we now further analyze the potential applications of the m6AScore system in different cancers. We first analyzed the association of Ms and m6AScore in different cancers, and the results showed that Ms and m6AScore were significantly associated in each cancer type in the pan-cancer database (Supplementary Figure S13C; Supplementary Table S19). We used cox proportional risk regression employing a univariate model to analyze the association between the m6AScore and the prognosis of each cancer. The results showed that m6AScore was a significant prognostic risk factor for most cancers, and we selected the TOP 10 displays in order of *p* value (Supplementary Figures S13D,E). The main clinically validated biomarkers reflecting the response to checkpoint blockade immunotherapy include: MSI, TMB, and inflammatory cell infiltration in TME (Razvan et al., 2018). The radar plot of the marker TMB showed a significant correlation between m6AScore and TMB for 15 of the 33 cancers (Figure 6M). Correlation analysis between markers MSI and m6AScore revealed a significant correlation for 26 of 33 cancers (Figure 6N). We also analyzed the correlation between PD-L1 and PD-1 expression values and m6AScore, and the results showed that 23 out of 33 cancers had a significant correlation between m6AScore and CD274 (Figure 6O); 20 out of 33 cancers had a significant correlation between m6AScore and CD8A (Figure 6P). Over the course of the entire checkpoint blockade immunotherapy marker analysis, trends were not uniform across cancers in the correlation of markers with m6AScore, with positive and negative correlations. This precisely demonstrated the reasonable and accurate design of our m6AScore system. Since each cancer is distinct in terms of immune infiltration of TME, different ICs and BP may be involved. Finally, we analyzed the correlation between the m6AScore and the ICs fraction for each cancer type, selecting a total of 54 ICs populations and performing a single-sample gene-set enrichment analysis for each cell population in a pan-cancer cohort of 11014 samples (Figure 6Q; Supplementary Table S20). PAAD and PCPG were found to be positively correlated with ICs, however, the trend of correlation was not uniform for other cancers. Most ICs were associated with the m6AScore in most cancers, which contains both pro- and anti-tumor ICs, suggesting that the effects of the individual Ms on immune function may not be uniform.

## 4 Discussion

In this study, we retrieved relevant data from the TCGA and CGGA databases for GBM and other cancers, including mRNA expression, post-translational modifications, molecular subtypes, DNA methylation, mutations, and relevant clinical parameters. Then based on Ms, we showed two different kinds of m6A methylation modification patterns. Moreover, by multi-omics analysis of Ms, we identified the association between these Ms in GBM tumorigenesis and distinct tumor subtypes. Through ssGSEA analysis, we obtained a better understanding of GBM with different modification patterns and identified a number of malignant hallmarks of tumors associated with a worse prognosis in the CL2 subgroup, including angiogenesis, DNA repair, coagulation, IL -6/JAK/STAT3 signaling, PI3K/AKT/MTOR signaling, and TGFβ signaling.

In recent years, several studies have demonstrated that Ms can affect GSCs, involving the promotion of stem cell self-renewal, induction of tumorigenesis, tumor cell proliferation and apoptosis, and resistance to adjuvant therapy (Dixit et al., 2021). The m6A writers are essential for the development of GBM, for example, the m6A methyltransferase METTL3 maintains its oncogenic effects by regulating the splicing factor NMD and selective splice isoform switch in GBM (Li et al., 2019). In addition, overexpression of the dominant-negative mutant METTL3 or silencing METTL3 inhibited the self-renewal and growth of GSCs (Li et al., 2019). In the reported studies, the m6A erasers have been shown to promote the development of GBM. For example, FTO not only promoted the occurrence of GBM but also induced GBM resistance to the alkylating agent temozolomide (TMZ), indicating that FTO may be a new target for GBM treatment (Xiao et al., 2020). Previously, YTHDC1 was the only m6A reader that has been proven to have an impact on GBM, and it affects the progress of GBM by binding to RNA (Li et al., 2019). A recent study demonstrated that the m6A reader YTHDF2 specifically stabilizes MYC mRNA in cancer stem cells and that YTHDF2 provides a therapeutic target to interfere with MYC signaling in GBM (Dixit et al., 2021). However, some scholars have presented a different view that some writers have anti-cancer properties (Dong and Cui, 2020). This further illustrates the necessity of our comprehensive research. In particular, revealing the relationship between different patterns of m6A modification and TME ICs infiltration and cancer stemness of GBM will help broaden our understanding of m6A modification and provide theoretical support for subsequent studies.

Some studies have reported the relationship between m6A methylation and immune infiltration, cancer stemness that cannot be explained by classical RNA degradation (Zhang et al., 2020). It has been demonstrated that METTL3 contributes to the activation of DCs and that the specific depletion of METTL3 in DCs leads to impaired phenotype and function of DCs, reduced expression of co-stimulatory molecules (CD40, CD80, and IL-12), and reduced ability to stimulate T cell responses (Wang et al., 2019). The homeostasis of T cells can be regulated by METTL3 through the IL-7/STAT5/SOCS pathway (Li et al., 2017). We further analyzed the TME ICs infiltration in both clusters and quantified the stemness index of individual tumors to determine the immune phenotypes of both clusters, with the CL1 subgroup showing an immune-activating differentiation phenotype, and the CL2 subgroup showing an immune desert dedifferentiation phenotype. It is noteworthy that although the immune excluded phenotype also revealed the presence of a large number of ICs, they did not penetrate the tumor cell parenchyma but were reserved in the stroma surrounding the tumor cell nests (Zhang et al., 2020). There are reports that the cellular stroma may be confined within the tumor envelope or possibly penetrate through the tumor, making it appear that the ICs are actually inside the tumor (Gajewski, 2015). METTL3 has been shown to be associated with the maintenance of stemness in GCSs and to mediate resistance to radiotherapy for GBM (Visvanathan et al., 2019). We identified hub genes and performed functional annotation to elucidate that m6A methylation modifications regulate potential mechanisms of cell stemness and immune phenotypes. In our study, inconsistent ratios of anti-tumor and pro-tumor ICs in individual tumor TME, disruption of oncogenic dedifferentiation phenotypes from distinct pathways, and dysregulation of different signaling pathways may be associated with m6A modification patterns. This study also confirmed that mRNA transcriptome differences between different m6A modification patterns were significantly correlated with stemness and immune-associated biological pathways. It is possible to consider these differentially expressed genes as m6A-associated signature genes. Moreover, Ms selectively consumed in TME can benefit patients receiving immunotherapy by reducing the infiltration of immunosuppressive cells (Tong et al., 2018). This also explains why our immune group A and stemness group A patients have a survival advantage, and confirms the accuracy of our calculation and clustering methods.

Quantification of m6A modification patterns in individual tumors is particularly important because of the individual heterogeneity of m6A modifications. For that, we analyzed Ms’ expression values, performed PCA analysis to construct m6A-associated gene signatures and built the m6AScore system. The m6A modification pattern characterized by immune-activating differentiation phenotype exhibited a lower m6Ascore, while the pattern characterized by immune desert dedifferentiation phenotype showed a higher m6Ascore. Reflecting individual methylation modification patterns, the m6AScore system could accurately stratify immune and stemness regulatory patterns that have different survival rates, molecular and immune characteristics. Our results confirmed that the m6AScore system could not only affect the prognosis of GBM patients with molecular subtypes, methylation status, 1p19q codeletion, and different modification patterns, but also independently predict the prognosis of GBM patients. EMT, as the process by which epithelial cells acquire mesenchymal characteristics, is related to tumor invasion, initiation, metastasis and resistance to treatment in many cancers (Paget, 1889). EMT can confer stem cell-like properties to cancer cells, driving stemness and tumorigenic features of the cells (Morel et al., 2008). EMT presenting TMZ resistance mechanisms were highly enriched in the m6AScore high group in our study. Interestingly, the majority of transcriptomic data from the database came from pre-EMT state primary tumors. However, there are also primary solid tumor cells that are not in a pre-EMT state, although other cells in this situation can still acquire mesenchymal features through additionally mutated accumulation and then spread and metastasize through the lymphatic and hematologic systems (Fabregat et al., 2016). In addition, it was reported that the EMT induced by TGF-β in hepatoma cells was related to changes in stem marker expression (Fernando et al., 2015). Our data also showed significant TGF-β enrichment in the high m6AScore subgroup, suggesting that this association also applies in GBM.

Novel immunotherapies represented by anti-PD-1 and PD-L1 (immune checkpoint blockade) are a developing and promising field. We observed a consistent association between anti-PD-L1 and anti-PD-1 treatment response and m6AScore with that between m6AScore and GBM, which we suggested may be due to the relatively high component of ICs infiltration in the m6AScore high group (Karachi et al., 2018). m6AScore system showed consistency in the immune and stemness patterns also suggesting that our system can act as a representative of the m6A modification patterns of individuals. Surprisingly, the determination regarding the immunophenotype was also well validated in the IMvigor210 cohort with a defined immunophenotype (Mariathasan et al., 2018). This demonstrated that our m6Ascore system is a trusted tool for comprehensive assessment of individual tumor m6A modification patterns, which can be used to determine tumor immune and stemness phenotypes and predict patient sensitivity to novel immunotherapies, and has potential for future clinical application as a pre-evaluation system prior to immunotherapy.

Applying the m6AScore system in other cancers treated with novel immunotherapy, the low m6AScore group also presented a significant survival advantage. It can be demonstrated that the m6AScore also represents the malignancy and aggressiveness of the cancer to some extent. Almost all Ms showed significant differences in expression between cancer and normal tissues. In tumors, the correlation between cancer cell stemness, immune signatures and m6AScore may suggest that both of them are influenced by m6A methylation, leading to uncontrolled immune dysregulation and dedifferentiation defined by origin structure loss. Integrated with several biomarkers, including TMB, MSI status, PD-L1 expression (CD274), PD-1 expression (CD8A), the m6A gene signature may be a more effective predictive strategy for immunotherapy. In the study of a pan-cancer cohort, the relationship between the m6AScore system and the different phenotypes of multiple tumors probably reflects the TME immune infiltration specificity, differences in immune checkpoint expression, BP diversity, and cancer cell stemness specificity. Our results also demonstrated that high m6AScore was associated with mesenchymal subtype, Immunity Group B, Stemness Group B, high infiltration of ICs, low sensitivity to immunotherapy. Overall, the m6Ascore system has the potential to be applied to comprehensively assess m6A methylation modification patterns, TME cell infiltration and stemness characteristics in individual patients to further define the immune phenotype and develop a more reasonable treatment plan. Studies in pan-cancer cohorts also confirmed that the m6AScore systerm can be applied to multiple cancers, with the corresponding m6AScore representing m6A methylation modifications, classifying tumors according to the degree of modification and providing reasonable predictions about patient treatment sensitivity and prognosis.

## 5 Conclusion

In conclusion, our work demonstrated the relevant regulatory mechanisms of m6A methylation modifications on GBM and other tumors TME ICs infiltration, stemness, and BP. The clustering based on the expression profiles of Ms confirmed that differences in m6A modification patterns are important factors contributing to the complexity and heterogeneity of individual cell stemness and TME ICs infiltration. A comprehensive assessment of tumor m6A modification patterns will help to improve our understanding of the characteristics of cancer cell stemness and TME ICs infiltration and guide the development of more effective immunotherapeutic approaches. Our m6AScore system can also be used to comprehensively assess m6A modification patterns as well as patient sensitivity and survival benefit to various therapies. Our results will facilitate the development of diagnostic tools to quantify cancer methylation modifications in individual tumors, with possible future applications to predict cancer recurrence, the selection of therapies, and the identification of new biomarkers of therapeutic response.

## Data Availability

The datasets presented in this study can be found in online repositories. The names of the repository/repositories and accession number(s) can be found in the article/Supplementary Material.

## References

[B1] Boccaletto P. Machnicka M. A. Purta E. Piątkowski P. Bagiński B. Wirecki T. K. (2018). MODOMICS: a Database of RNA Modification Pathways. 2017 Update. Nucleic Acids Res. 46, D303–D307. 10.1093/nar/gkx1030 29106616 PMC5753262

[B2] Chen X. Y. Zhang J. Zhu J. S. (2019). The Role of m6A RNA Methylation in Human Cancer. Mol. Cancer 18, 103–109. 10.1186/s12943-019-1033-z 31142332 PMC6540575

[B3] Cloughesy T. F. Mochizuki A. Y. Orpilla J. R. Hugo W. Lee A. H. Davidson T. B. (2019). Neoadjuvant Anti-PD-1 Immunotherapy Promotes a Survival Benefit with Intratumoral and Systemic Immune Responses in Recurrent Glioblastoma. Nat. Med. 25, 477–486. 10.1038/s41591-018-0337-7.Neoadjuvant 30742122 PMC6408961

[B4] Cui Q. Shi H. Ye P. Li L. Qu Q. Sun G. (2017). m 6 A RNA Methylation Regulates the Self-Renewal and Tumorigenesis of Glioblastoma Stem Cells. Cel Rep. 18, 2622–2634. 10.1016/j.celrep.2017.02.059 PMC5479356 28297667

[B5] Dixit D. Prager B. C. Gimple R. C. Poh H. X. Wang Y. Wu Q. (2021). The RNA m6A Reader YTHDF2 Maintains Oncogene Expression and Is a Targetable Dependency in Glioblastoma Stem Cells. Cancer Discov. 11, 480–499. 10.1158/2159-8290.cd-20-0331 33023892 PMC8110214

[B6] Dominissini D. Moshitch-Moshkovitz S. Schwartz S. Salmon-Divon M. Ungar L. Osenberg S. (2012). Topology of the Human and Mouse m6A RNA Methylomes Revealed by m6A-Seq. Nature 485, 201–206. 10.1038/nature11112 22575960

[B7] Dong Z. Cui H. (2020). The Emerging Roles of RNA Modifications in Glioblastoma. Cancers 12, 736. 10.3390/cancers12030736 PMC7140112 32244981

[B8] Fabregat I. Malfettone A. Soukupova J. (2016). New Insights into the Crossroads between EMT and Stemness in the Context of Cancer. Jcm 5, 37. 10.3390/jcm5030037 PMC4810108 26985909

[B9] Fernando J. Malfettone A. Cepeda E. B. Vilarrasa-Blasi R. Bertran E. Raimondi G. (2015). A Mesenchymal-like Phenotype and Expression of CD44 Predict Lack of Apoptotic Response to Sorafenib in Liver Tumor Cells. Int. J. Cancer 136, E161–E172. 10.1002/ijc.29097 25053293

[B10] Gajewski T. F. (2015). The Next Hurdle in Cancer Immunotherapy: Overcoming the Non-T-cell-inflamed Tumor Microenvironment. Semin. Oncol. 42, 663–671. Elsevier. 10.1053/j.seminoncol.2015.05.011 26320069 PMC4555998

[B11] Geula S. Moshitch-Moshkovitz S. Dominissini D. Mansour A. A. Kol N. Salmon-Divon M. (2015). m 6 A mRNA Methylation Facilitates Resolution of Naïve Pluripotency toward Differentiation. Science 347, 1002–1006. 10.1126/science.1261417 25569111

[B13] He J. Zhou M. Yin J. Wan J. Chu J. Jia J. (2021). METTL3 Restrains Papillary Thyroid Cancer Progression via m6A/c-Rel/IL-8-mediated Neutrophil Infiltration. Mol. Ther. 29, 1821–1837. 10.1016/j.ymthe.2021.01.019 33484966 PMC8116572

[B44] He J. Zhu S. Liang X. Zhang Q. Luo X. Liu C. (2021). LncRNA as a Multifunctional Regulator in Cancer Multi-Drug Resistance. Mol. Biol. Rep. 48, 1–15. 10.1007/s11033-021-06603-7 34333735

[B14] Hegde P. S. Karanikas V. Evers S. (2016). The where, the when, and the How of Immune Monitoring for Cancer Immunotherapies in the Era of Checkpoint Inhibition. Clin. Cancer Res. 22, 1865–1874. 10.1158/1078-0432.CCR-15-1507 27084740

[B15] Hernández-Vega A. M. Del Moral-Morales A. Zamora-Sánchez C. J. Piña-Medina A. G. González-Arenas A. Camacho-Arroyo I. (2020). Estradiol Induces Epithelial to Mesenchymal Transition of Human Glioblastoma Cells. Cells 9, 1930. 10.3390/cells9091930 PMC7564468 32825553

[B17] Hugo W. Zaretsky J. M. Sun L. Song C. Moreno B. H. Hu-Lieskovan S. (2016). Genomic and Transcriptomic Features of Response to Anti-PD-1 Therapy in Metastatic Melanoma. Cell 165, 35–44. 10.1016/j.cell.2016.02.065 26997480 PMC4808437

[B18] Karachi A. Dastmalchi F. Mitchell D. A. Rahman M. (2018). Temozolomide for Immunomodulation in the Treatment of Glioblastoma. Neuro. Oncol. 20, 1566–1572. 10.1093/neuonc/noy072 29733389 PMC6231207

[B19] Kwok C. T. Marshall A. D. Rasko J. E. Wong J. J. (2017). Genetic Alterations of m6A Regulators Predict Poorer Survival in Acute Myeloid Leukemia. J. Hematol. Oncol. 10, 39. 10.1186/s13045-017-0410-6 28153030 PMC5290707

[B20] Lee A. H. Sun L. Mochizuki A. Y. Reynoso J. G. Orpilla J. Chow F. (2021). Neoadjuvant PD-1 Blockade Induces T Cell and cDC1 Activation but Fails to Overcome the Immunosuppressive Tumor Associated Macrophages in Recurrent Glioblastoma. Nat. Commun. 12, 1–16. 10.1038/s41467-021-26940-2 34836966 PMC8626557

[B21] Li F. Yi Y. Miao Y. Long W. Long T. Chen S. (2019). N6-methyladenosine Modulates Nonsense-Mediated mRNA Decay in Human Glioblastoma. Cancer Res. 79, 5785–5798. 10.1158/0008-5472.can-18-2868 31530567 PMC7360104

[B16] Li H. Tong J. Zhu S. Batista B. J. Duffy E. E. Zhao J. (2017). m6A mRNA Methylation Controls T Cell Homeostasis by Targeting the IL-7/STAT5/SOCS Pathways. Nature 17, 338–342. 10.1038/nature23450 PMC5729908 28792938

[B22] Li Z. Weng H. Su R. Weng X. Zuo Z. Li C. (2017). FTO Plays an Oncogenic Role in Acute Myeloid Leukemia as a N 6 -Methyladenosine RNA Demethylase. Cancer Cell 31, 127–141. 10.1016/j.ccell.2016.11.017 28017614 PMC5234852

[B23] Ma J. Z. Yang F. Zhou C. C. Liu F. Yuan J. H. Wang F. (2017). METTL14 Suppresses the Metastatic Potential of Hepatocellular Carcinoma by Modulating N 6 ‐methyladenosine‐dependent Primary MicroRNA Processing. Hepatology 65, 529–543. 10.1002/hep.28885 27774652

[B24] Ma Z. Ji J. (2020). N6-methyladenosine (m6A) RNA Modification in Cancer Stem Cells. Stem Cells 38, 1511–1519. 10.1002/stem.3279 32985068

[B25] Malta T. M. Sokolov A. Gentles A. J. Burzykowski T. Poisson L. Weinstein J. N. (2019). Machine Learning Identifies Stemness Features Associated with Oncogenic Dedifferentiation. Cell 173, 338. 10.1016/j.cell.2018.03.034 PMC5902191 29625051

[B26] Mariathasan S. Turley S. J. Nickles D. Castiglioni A. Yuen K. Wang Y. (2018). TGFβ Attenuates Tumour Response to PD-L1 Blockade by Contributing to Exclusion of T Cells. Nature 554, 544–548. 10.1038/nature25501 29443960 PMC6028240

[B27] McCubrey J. A. Rakus D. Gizak A. Steelman L. S. Abrams S. L. Lertpiriyapong K. (2016). Effects of Mutations in Wnt/β-Catenin, Hedgehog, Notch and PI3K Pathways on GSK-3 Activity-Diverse Effects on Cell Growth, Metabolism and Cancer. Biochim. Biophys. Acta (Bba) - Mol. Cel Res. 1863, 2942–2976. 10.1016/j.bbamcr.2016.09.004 27612668

[B28] Morel A.-P. Lièvre M. Thomas C. Hinkal G. Ansieau S. Puisieux A. (2008). Generation of Breast Cancer Stem Cells through Epithelial-Mesenchymal Transition. PLoS One 3, e2888. 10.1371/journal.pone.0002888 18682804 PMC2492808

[B29] Nusrat M. (2020). Response to Anti-PD-1 in Microsatellite-Stable Colorectal Cancer: A STAT Need. Clin. Cancer Res. 26, 5775–5777. 10.1158/1078-0432.CCR-20-2901 32958701

[B30] Ott P. A. Hu-Lieskovan S. Chmielowski B. Govindan R. Naing A. Bhardwaj N. (2020). A Phase Ib Trial of Personalized Neoantigen Therapy Plus Anti-PD-1 in Patients with Advanced Melanoma, Non-small Cell Lung Cancer, or Bladder Cancer. Cell 183, 347–362. 10.1016/j.cell.2020.08.053 33064988

[B31] Paget S. (1889). The Distribution of Secondary Growths in Cancer of the Breast. The Lancet 133, 571–573. 10.1016/s0140-6736(00)49915-0 2673568

[B32] Pan Y. Xiao K. Li Y. Li Y. Liu Q. (2021a). RNA N6-Methyladenosine Regulator-Mediated Methylation Modifications Pattern and Immune Infiltration Features in Glioblastoma. Front. Oncol. 11, 1–15. 10.3389/fonc.2021.632934 PMC7947873 33718217

[B33] Pan Y. Zhao S. Chen F. (2021b). The Potential Value of Dequalinium Chloride in the Treatment of Cancer: Focus on Malignant Glioma. Clin. Exp. Pharmacol. Physiol. 48, 445–454. 10.1111/1440-1681.13466 33496065

[B34] Qin Y. Li L. Luo E. Hou J. Yan G. Wang D. (2020). Role of m6A RNA Methylation in Cardiovascular Disease (Review). Int. J. Mol. Med. 46, 1958–1972. 10.3892/ijmm.2020.4746 33125109 PMC7595665

[B35] Razvan C. Robin M. Mark A. Andrew A. Erin M. Jennifer Y. (2018). Pan-tumor Genomic Biomarkers for PD-1 Checkpoint Blockade–Based Immunotherapy. Science 80-362, 6411. 10.1126/science.aar3593 PMC6718162 30309915

[B36] Roignant J.-Y. Soller M. (2017). m 6 A in mRNA: An Ancient Mechanism for Fine-Tuning Gene Expression. Trends Genet. 33, 380–390. 10.1016/j.tig.2017.04.003 28499622

[B37] Roundtree I. A. Evans M. E. Pan T. He C. (2017). Dynamic RNA Modifications in Gene Expression Regulation. Cell 169, 1187–1200. 10.1016/j.cell.2017.05.045 28622506 PMC5657247

[B12] Salmaninejad A. Valilou S. F. Shabgah A. G. Aslani S. Alimardani M. Pasdar A. (2019). PD-1/PD-L1 Pathway: Basic Biology and Role in Cancer Immunotherapy. J. Cell Physiol. 234, 16824–16837. 10.1002/jcp.28358 30784085

[B38] Sanchez-Vega F. Mina M. Marra M. A. (2019). Pathways, Oncogenic Signaling Cancer, the Atlas, Genome. Cell 173, 321–337. 10.1016/j.cell.2018.03.035 PMC6070353 29625050

[B39] Sotiriou C. Wirapati P. Loi S. Harris A. Fox S. Smeds J. (2006). Gene Expression Profiling in Breast Cancer: Understanding the Molecular Basis of Histologic Grade to Improve Prognosis. J. Natl. Cancer Inst. 98, 262–272. 10.1093/jnci/djj052 16478745

[B40] Subramanian A. Tamayo P. Mootha V. K. Mukherjee S. Ebert B. L. Gillette M. A. (2005). Gene Set Enrichment Analysis: A Knowledge-Based Approach for Interpreting Genome-wide Expression Profiles. Proc. Natl Acad. Sci. USA 102, 15545–15550. 10.1073/pnas.0506580102 16199517 PMC1239896

[B41] Tang R. Zhang Y. Liang C. Xu J. Meng Q. Hua J. (2020). The Role of m6A-Related Genes in the Prognosis and Immune Microenvironment of Pancreatic Adenocarcinoma. PeerJ 8, e9602. 10.7717/peerj.9602 33062408 PMC7528816

[B42] Tong J. Cao G. Zhang T. Sefik E. Amezcua Vesely M. C. Broughton J. P. (2018). m6A mRNA Methylation Sustains Treg Suppressive Functions. Cell Res 28, 253–256. 10.1038/cr.2018.7 29303144 PMC5799823

[B43] Visvanathan A. Patil V. Abdulla S. Hoheisel J. Somasundaram K. (2019). N6-Methyladenosine Landscape of Glioma Stem-like Cells: METTL3 Is Essential for the Expression of Actively Transcribed Genes and Sustenance of the Oncogenic Signaling. Genes 10, 141. 10.3390/genes10020141 PMC6410051 30781903

[B45] Vu L. P. Pickering B. F. Cheng Y. Zaccara S. Nguyen D. Minuesa G. (2017). The N6-Methyladenosine (m6A)-Forming Enzyme METTL3 Controls Myeloid Differentiation of normal Hematopoietic and Leukemia Cells. Nat. Med. 23, 1369–1376. 10.1038/nm.4416 28920958 PMC5677536

[B46] Wang H. Hu X. Huang M. Liu J. Gu Y. Ma L. (2019). Mettl3-mediated mRNA m6A Methylation Promotes Dendritic Cell Activation. Nat. Commun. 10, 1898. 10.1038/s41467-019-09903-6 31015515 PMC6478715

[B47] Xiao L. Li X. Mu Z. Zhou J. Zhou P. Xie C. (2020). FTO Inhibition Enhances the Antitumor Effect of Temozolomide by Targeting MYC-miR-155/23a Cluster-MXI1 Feedback Circuit in Glioma. Cancer Res. 80, 3945–3958. 10.1158/0008-5472.CAN-20-0132 32680921

[B48] Yang S. Wei J. Cui Y.-H. Park G. Shah P. Deng Y. (2019). m6A mRNA Demethylase FTO Regulates Melanoma Tumorigenicity and Response to Anti-PD-1 Blockade. Nat. Commun. 10, 2782. 10.1038/s41467-019-10669-0 31239444 PMC6592937

[B49] Yang Y. Hsu P. J. Chen Y.-S. Yang Y.-G. (2018). Dynamic Transcriptomic m6A Decoration: Writers, Erasers, Readers and Functions in RNA Metabolism. Cel Res 28, 616–624. 10.1038/s41422-018-0040-8 PMC5993786 29789545

[B50] Yu Y. Yang B. Yu J. Zhao G. Chen F. (2020). Dequalinium Chloride Inhibits the Growth of Human Glioma Cells *In Vitro* and Vivo: a Study on Molecular Mechanism and Potential Targeted Agents. Acta Neurochir 162, 1683–1690. 10.1007/s00701-020-04401-x 32410120

[B51] Zepecki J. P. Karambizi D. Fajardo J. E. Snyder K. M. Guetta-Terrier C. Tang O. Y. (2021). miRNA-Mediated Loss of m6A Increases Nascent Translation in Glioblastoma. Plos Genet. 17, e1009086. 10.1371/journal.pgen.1009086 33684100 PMC7971852

[B52] Zhang B. Wu Q. Li B. Wang D. Wang L. Zhou Y. L. (2020). M6A Regulator-Mediated Methylation Modification Patterns and Tumor Microenvironment Infiltration Characterization in Gastric Cancer. Mol. Cancer 19, 53. 10.1186/s12943-020-01170-0 32164750 PMC7066851

