# Supplementary material for: The Role of m6A Regulator-Mediated Methylation Modification and Tumor Microenvironment Infiltration in Glioblastoma Multiforme
Source: Front Cell Dev Biol. 2022 Feb 21;10:842835. doi: 10.3389/fcell.2022.842835 (PMC8898963; doi:10.3389/fcell.2022.842835)
Supplement: Supplementary file 15 [file DataSheet1.docx]

Figure S1. Overview of the work and Expression correlation of m6A regulators.

A. Overview of our work. B. The expression of 21 m6A regulators between primary tissues and recurrence tissues in CGGA-GBM cohort. C. The expression of 21 m6A regulators between primary tissues and recurrence tissues in TCGA-GBM cohort. D-G. Difference in the expression value of readers (D) and writers (E) between low and high FTO (eraser) expression subgroups in CGGA-GBM cohort. Difference in the expression value of readers (F) and writers (G) between low and high ALKBH5 (eraser) expression groups in CGGA-GBM cohort. The upper and lower ends of the boxes represented an interquartile range of values. The lines in the boxes represented median value, and dots showed outliers. The asterisks represented the statistical p-value. (*P < 0.05; **P < 0.01; ***P < 0.001, ns, no significant).

Figure S2. Expression correlation and prognostic value of m6A regulators.

A-D. Difference in the expression value of readers (A) and writers (B) between low and high FTO (eraser) expression subgroups in TCGA-GBM cohort. Difference in the expression value of readers (C) and writers (D) between low and high ALKBH5 (eraser) expression subgroups in TCGA-GBM cohort. E-F. Univariate Cox regression (E) and multivariate Cox regression (F) analyzed the association between 21 m6A regulators and overall survival in TCGA-GBM combined CGGA-GBM cohort. Hazard ratio >1 represented risk factors for survival and hazard ratio <1 represented protective factors for survival. G-H. Correlation between expression value of m6A regulator and immune cell fraction and immune signatures, the immune cell fraction of m6A regulators (G) and the immune signatures of m6A regulators (H) in the TCGA-GBM combined CGGA-GBM cohort. The heat map shows the correlations, with red representing positive correlation, and blue representing negative correlation. The upper and lower ends of the boxes represented an interquartile range of values. The lines in the boxes represented median value, and dots showed outliers. The asterisks represented the statistical p-value. (*P < 0.05; **P < 0.01; ***P < 0.001, ns, no significant).

Figure S3. The expression of m6A regulators between TCGA subtypes.

ALKBH5 (p=0.075), CBLL1 (p=0.041), ELAVL1 (p<0.001), FMR1 (p=0.031), FTO (p<0.001), HNRNPA2B1 (p<0.001), HNRNPC (p=0.06), IGF2BP1 (p=0.29), LRPPRC (p=0.05), METTL3 (p<0.001), METTL14 (p=0.044), RBM15 (p=0.015), RBM15B (p=0.0082), WTAP (p=0.44), YTHDC1 (p<0.001), YTHDC2 (p=0.0076), YTHDF1 (p<0.001), YTHDF2 (p=0.61), YTHDF3 (p=0.61), ZC3H13 (p=0.018).

Figure S4. Annotation of classification functions determined by consensus clustering analysis.

A. The relative change in the area under the cumulative distribution function (CDF) curve for k = 2-10 in the CGGA-GBM cohort. B. The relative change in the area under the CDF curve for k = 2-10 in the TCGA-GBM cohort. C. Consensus clustering matrix for k = 3 in the CGGA-GBM cohort. D. Consensus clustering matrix for k = 3 in the TCGA-GBM cohort. E. The proportions of IDH molecular subtypes in the CL1 or CL2 subgroups. F. The proportions of TCGA subtypes in the CL1 or CL2 subgroups. G. Functional annotation for m6A-related genes using GO enrichment analysis in CGGA-GBM cohort. H. Functional annotation for m6A-related genes using KEGG enrichment analysis in CGGA-GBM cohort. The size of the barplots represented the number of genes enriched. I-O. GSEA revealed that genes with higher expression in the CL2 subgroup were enriched for hallmarks of malignant tumors in the CGGA-GBM cohort, Angiogenesis (I), DNA repair (J), Coagulation (K), IL6-JAK-STATS signaling (L), Kras signaling (M), PI3k-AKT-MTOR signaling (N), TGFβ (O). P. The enrichment fraction differences of immune cells between CL1 and CL2 subgroups in the CGGA-GBM cohort. Q. The enrichment differences of biological processes between CL1 and CL2 subgroups in the CGGA-GBM cohort. R. The enrichment differences of biological processes between CL1 and CL2 subgroups in the TCGA-GBM cohort. The upper and lower ends of the boxes represented an interquartile range of values. The lines in the boxes represented median value, and dots showed outliers. The asterisks represented the statistical p-value. (*P < 0.05; **P < 0.01; ***P < 0.001, ns, no significant).

Figure S5. Characterization of immuno-infiltration and stemness in samples with specific m6A modification patterns, and tumor somatic mutation.

A-C. Correlation of samples between infiltration of cell types executing pro-tumor, immune-suppressive functions (pDC, Neutrophil, CD56dimNK, TAM, imDC, Th2, MDSC, and Treg) and cell types executing anti-tumor immunity (NKT, TemCD4, TemCD8, ActCD4, ActCD8, Th1, Th17, ActDC, TcmCD4, TcmCD8, CD56briNK and NK). A. All samples in the CGGA-GBM dataset, B. CL1 subgroup samples in the CGGA-GBM dataset, C. CL2 subgroup samples in the CGGA-GBM dataset. D. Differences in the expression of stimulator genes in the CL1 and CL2 subgroups of CGGA-GBM cohort. E. Differences in the expression of stimulator genes in the CL1 and CL2 subgroups of CGGA-GBM cohort. F. Differences in the expression of major histocompatibility complex (MHC) genes in the CL1 and CL2 subgroups of CGGA-GBM cohort. G. Differences in the expression of m6A regulators in the EREG-mRNAsi-high and EREG-mRNAsi-low subgroups in TCGA-GBM cohort. H. Differences in the expression of m6A regulators in the EREG-mDNAsi-high and EREG-mDNAsi-low subgroups in TCGA-GBM cohort. I. The waterfall plot of tumor somatic mutation was established by those with the CL1 subgroup in TCGA-GBM cohort. J. The waterfall plot of tumor somatic mutation was established by those with the CL2 subgroup in TCGA-GBM cohort. Each column represented individual patients. The upper barplot showed tumor mutational burden, The number on the right indicated the mutation frequency in each gene. The right barplot showed the proportion of each variant type. The upper and lower ends of the boxes represented an interquartile range of values. The lines in the boxes represented median value, and dots showed outliers. The asterisks represented the statistical p-value. (*P < 0.05; **P < 0.01; ***P < 0.001, ns, no significant).

Figure S6. Hub genes and their functional annotation.

A. Analysis of network topology for various soft-thresholding powers in scale independence and mean connectivity. B. Analysis of network topology for various soft-thresholding powers in scale independence and mean connectivity. C-E. Scatter plot of module eigengenes in all modules with mRNAsi, C. grey, blue, turquoise, red, D. brown, pink, purple, greenyellow, E. green, black, magenta, yellow. F-H. Scatter plot of module eigengenes in all modules with ESTIMATE score, F. grey, blue, turquoise, red, G. brown, pink, purple, greenyellow, H. green, black, magenta, yellow.

Figure S7. Characteristics of potential traits in m6A-related phenotypes.

A-C. Scatter plot of module eigengenes in all modules with mDNAsi, A. grey, blue, turquoise, red, B. brown, pink, purple, greenyellow, C. green, black, magenta, yellow. D-E. consensus clustering analysis based on the 159 immune phenotype-related genes, consensus clustering matrix for k = 3 (D)，k=4 (E) in the TCGA-GBM cohort. F-I. Consensus clustering analysis based on the 159 immune phenotype-related genes, consensus clustering matrix for k = 2 (F), k=3 (G)，k=4 (H) in the TCGA-GBM cohort. Relative change in area under the CDF curve for k = 2-10 in the TCGA-GBM cohort (I). J. The expression of m6A regulators between 2 immunity clusters in TCGA-GBM cohort. K. The expression of m6A regulators between 2 stemness clusters in TCGA-GBM cohort. L. The enrichment of typical stemness markers between the 2 stemness clusters in TCGA-GBM cohort. The upper and lower ends of the boxes represented an interquartile range of values. The lines in the boxes represented median value, and dots showed outliers. The asterisks represented the statistical p-value. (*P < 0.05; **P < 0.01; ***P < 0.001, ns, no significant).

Figure S8. Differences in the expression of genes among the Stemness clusters and Immunity clusters, unsupervised clustering process based on methylation sites.

A. The enrichment of infiltrating immune cells and stemness indices in the 2 immunity clusters in TCGA-GBM cohort. B. The enrichment of infiltrating immune cells and stemness indices in the 2 stemness clusters in TCGA-GBM cohort. C. The expression of MHC genes among Immunity clusters in TCGA-GBM cohort. D. The expression of MHC genes among stemness clusters in TCGA-GBM cohort. E. The expression of stimulator genes among Immunity clusters in TCGA-GBM cohort. F. The expression of stimulator genes among stemness clusters in TCGA-GBM cohort. The upper and lower ends of the boxes represented an interquartile range of values. The lines in the boxes represented median value, and dots showed outliers. The asterisks represented the statistical p-value. (*P < 0.05; **P < 0.01; ***P < 0.001, ns, no significant).

Figure s9. Effects of m6A modification in antitumor therapy and tumor somatic mutation.

A. Heatmap of methylation clusters with clinical and molecular pathological parameters. B-D. Regression coefficient profiles of identified m6A regulators in the TCGA-GBM cohort, survival analyses for subgroup patients stratified by both m6AScore and IDH (B), and MGMT (C), and X1p19q (D) using Kaplan–Meier curves in the Log-rank test. E. Survival analyses for subgroup patients stratified by both m6AScore and CL1, CL2 in TCGA-GBM cohort using Kaplan–Meier curves in the Log-rank test. F. Survival analyses for low-m6AScore and high-m6AScore groups in TCGA-GBM combined CGGA-GBM cohort using Kaplan–Meier in Log-rank test. G. Differences in the expression values of m6A regulators among low- and high-m6AScore groups in the TCGA-GBM combined CGGA-GBM cohort. H. Differences in 10 oncogenic pathways between the low-m6AScore and high-m6AScore groups in the TCGA-GBM combined CGGA-GBM cohort. I-J. Correlation of high-m6AScore (I), and low-m6AScore (J) between infiltration of cell types executing pro-tumor, immune-suppressive functions (pDC, Neutrophil, CD56dimNK, TAM, imDC, Th2, MDSC, and Treg) and cell types executing anti-tumor immunity (NKT, TemCD4, TemCD8, ActCD4, ActCD8, Th1, Th17, ActDC, TcmCD4, TcmCD8, CD56briNK and NK). R coefficient of Pearson‘s correlation. The shaded area represents 95% confidence interval. K-L. The waterfall plot of tumor somatic mutation was established by those with high m6Ascore (K) and low m6Ascore (L). The upper and lower ends of the boxes represented an interquartile range of values. The lines in the boxes represented the median value, and the dots showed outliers. The asterisks represented the statistical P-value (*P < 0.05; **P < 0.01; ***P < 0.001, ns, no significant).

Figure S10. The construction process of m6AScore system.

A-C. Regression coefficient profiles of identified m6A regulators in the CGGA-GBM cohort, survival analyses for subgroup patients stratified by both m6AScore and IDH (A), MGMT (B), X1p19q (C) using Kaplan–Meier curves in the Log-rank test. D. Survival analyses for subgroup patients stratified by both m6AScore and CL1, CL2 subgroups in CGGA-GBM cohort using Kaplan–Meier curves in the Log-rank test. E-H. Survival analysis of low-m6AScore group and high-m6AScore group with different GBM subtypes in the TCGA-GBM cohort in the Log-rank test, including classical (E), proneural (F), neural (G), mesenchymal (H). I-K. Differences in m6AScore among TCGA-GBM subtype (I), stemness clusters (J), immunity clusters (K) in the TCGA-GBM cohort. L. Abundance differences BP between low-m6AScore and high-m6AScore subgroups in the TCGA-GBM cohort. M. Abundance differences BP between low-m6AScore and high-m6AScore subgroups in the CGGA-GBM cohort. N. Abundance differences in immune cells and pathways between low-m6AScore and high-m6AScore subgroups in the TCGA-GBM cohort. O. Abundance differences in immune cells and pathways between low-m6AScore and high-m6AScore subgroups in the CGGA-GBM cohort. The upper and lower ends of the boxes represented an interquartile range of values. The lines in the boxes represented median value, and dots showed outliers. The asterisks represented the statistical p-value. (*P < 0.05; **P < 0.01; ***P < 0.001, ns, no significant).

Figure S11. The role of the m6AScore in anti-PD-1/L1 immunotherapy.

A. The expression of m6A regulators between neoadjuvant and adjuvant therapy (GSE121810). B. The negative correlation between the m6AScore and tumor mutational burden. C. Differences in 10 oncogenic pathways between low-m6AScore and high-m6AScore subgroups in the IMvigor210CoreBiologies cohort (anti-PD1). D. Differences in 5 biological pathways between low-m6AScore and high-m6AScore subgroups in the IMvigor210CoreBiologies cohort (anti-PD1). E. Differences in 10 oncogenic pathways between low-m6AScore and high-m6AScore subgroups in the GBM cohort (anti-PD1). F. Differences in 5 biological pathways between low-m6AScore and high-m6AScore subgroups in the GBM cohort (anti-PD1). G. Differences in pro- and anti-tumor immune cells between low-m6AScore and high-m6AScore subgroups in the GBM cohort (anti-PD1). H. Heatmap and clinicopathologic features of the low-m6AScore and high-m6AScore. The upper and lower ends of the boxes represented an interquartile range of values. The lines in the boxes represented median value, and dots showed outliers. The asterisks represented the statistical p-value. (*P < 0.05; **P < 0.01; ***P < 0.001, ns, no significant).

Figure S12. Pan-cancer genetic and expression alterations of m6A regulators.

A. Heatmaps of expression levels of 21 m6A RNA methylation regulators in the Pan-cancer cohort, 11014 samples. B. The expression of 21 m6A regulators between normal tissues and tumor tissues in the Pan-cancer cohort. C-D. The correlations between immune-related genes CD274 (C), CD8A (D) and m6A regulators. The upper and lower ends of the boxes represented an interquartile range of values. The lines in the boxes represented median value, and dots showed outliers. The asterisks represented the statistical p-value. (*P < 0.05; **P < 0.01; ***P < 0.001, ns, no significant).

Figure S13. m6AScore in anti-PD-1/L1 immunotherapy and pan-cancer.

A-B. The correlations between immune-related genes GZMA(A), PRF1(B) and m6A regulators. C. Correlation between m6AScore and the expression value of m6A regulators for each cancer type. D-E. The prognosis-free interval (PF) (D) and overall survival (OS) (E) analyses for the m6AScore in TCGA cancer types using a univariate Cox regression model.
